# Dynamic Interconversions of Single Molecules Probed by Recognition Tunneling at Cucurbit[7]uril‐Functionalized Supramolecular Junctions

**DOI:** 10.1002/anie.202203830

**Published:** 2022-05-05

**Authors:** Bohuai Xiao, Suhang He, Mingjun Sun, Jianghao Zhou, Zhiye Wang, Yunchuan Li, Simin Liu, Werner M. Nau, Shuai Chang

**Affiliations:** ^1^ The State Key Laboratory of Refractories and Metallurgy the Institute of Advanced Materials and Nanotechnology College of Materials and Metallurgy Wuhan University of Science and Technology Wuhan Hubei 430081 China; ^2^ School of Science Jacobs University Bremen 28759 Bremen Germany; ^3^ The State Key Laboratory of Refractories and Metallurgy School of Chemistry and Chemical Engineering Wuhan University of Science and Technology Wuhan Hubei 430081 China

**Keywords:** Host–Guest Complexes, Molecular Conductance, Molecular Electronics, Molecular Recognition, Single Molecule Spectroscopy

## Abstract

We introduce a versatile recognition tunneling technique using doubly cucurbit[7]uril‐functionalized electrodes to form supramolecular junctions that capture analytes dynamically by host–guest complexation. This results in characteristic changes in their single‐molecule conductance. For structurally related drug molecules (camptothecin, sanguinarine, chelerythrine, and berberine) and mixtures thereof, we observed distinct current switching signals related to their intrinsic conductance properties as well as pH‐dependent effects which can be traced back to their different states (protonated versus neutral). The conductance variation of a single molecule with pH shows a sigmoidal distribution, allowing us to extract a p*K*_a_ value for reversible protonation, which is consistent with the reported macroscopic results. The new electronic method allows the characterization of unmodified drug molecules and showcases the transfer of dynamic supramolecular chemistry principles to single molecules.

## Graphical abstract

Dynamic conversions of single molecules can be monitored at doubly cucurbit[7]uril‐functionalized supramolecular junctions. Through this intermolecular recognition tunneling technique, the molecular conductance of different analytes and analyte mixtures can be finger‐printed in dependence on pH and competitive salts, showcasing the first examples of direct as well as displacement titrations of single host–guest complexes at electronic junctions.

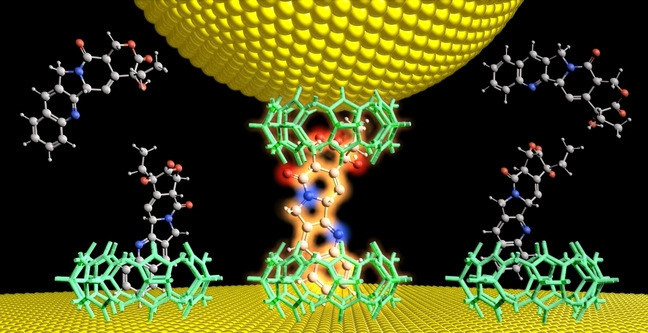

Electronic techniques based on recognition tunneling could eventually become important tools for studying physicochemical properties (p*K*_a_), probing chemical interconversions as well as intermolecular interactions (molecular recognition, affinities), and monitoring biological functionality (active states) at the single‐molecule level. Protonation, tautomerization, or hydration, in particular, are among the simplest aqueous processes that lead to large changes in physical, chemical, and biological properties and activities.^[1]^ For example, camptothecin (CPT), is an effective drug in the treatment of leukaemia, which owes its physiological activity to the lactone form (Scheme 1) that, however, predominates over the inactive carboxylate form only in acidic solution (pH<4).^[2]^ Sanguinarine (SA) and chelerythrine (CHE, Scheme 1), on the other hand, are natural benzophenanthridine alkaloids that exhibit anticancer, antimicrobial, and antifungal properties attributed to their iminium form, which is converted to the inactive alkanolamine form in basic conditions.^[3]^ While the corresponding molecular interconversions can be readily monitored by ensemble spectroscopic techniques, such as NMR, UV/Vis, and fluorescence measurements, the probing of a particular state of a single molecule presents a challenge not only from an analytical‐chemical but also from a conceptual point of view, as the interconversion of an inactive into an active drug molecule could eventually be followed near its biological target with spatial and temporal resolution.[^1a^, ^1b^]

**Scheme 1:**
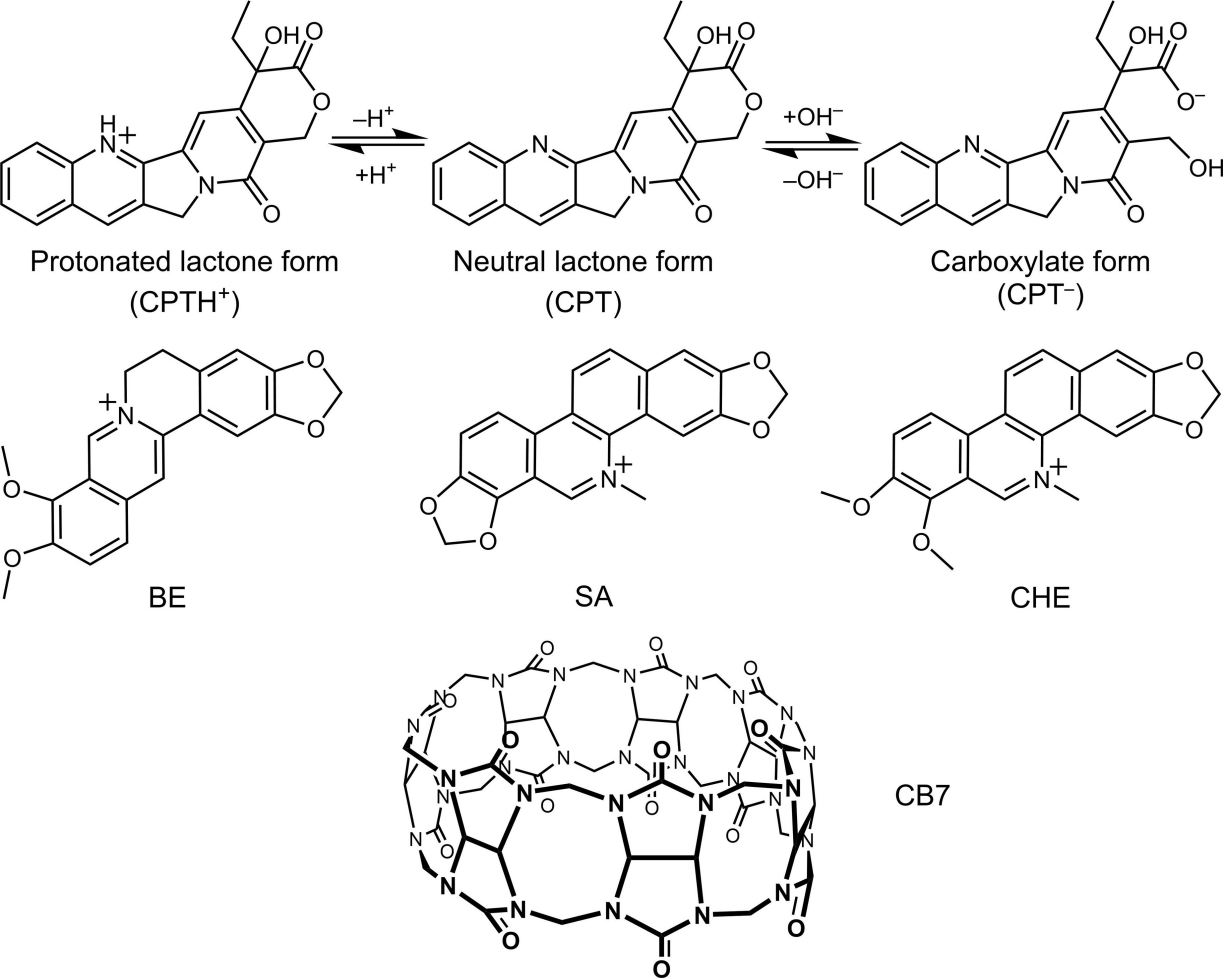
Top: pH‐dependent interconversion between the active lactone (left and middle) and the inactive carboxylate (right) states of camptothecin (CPT). Middle: Chemical structure of berberine (BE) and of the cationic forms of sanguinarine (SA) and chelerythrine (CHE). Bottom: Chemical structure of cucurbit[7]uril (CB7).

The present work builds upon the known propensity of the macrocyclic host cucurbit[7]uril (CB7) to attach to gold surfaces,^[4]^ where chemically unmodified analytes can bind reversibly inside the CB7 cavity by a molecular recognition process. While previous studies have focused on analyte detection,^[5]^ we now advance a “supramolecular‐junction” based recognition tunneling technique that allows access to dynamic host–guest titrations with single molecules. We show that this approach can be used to differentiate between different molecular states, to obtain physical properties (such as p*K*_a_), and to determine host–guest binding affinities, all by measuring single‐molecule electronic properties. We performed measurements with the pH‐sensitive drug molecules CPT, SA, and CHE and used berberine (BE) as a control, because it is not being protonated or deprotonated in the investigated pH range.^[6]^

Figure 1a shows the schematic layout of the CB7‐assisted recognition tunneling measurement. An electrochemically etched gold probe is placed above a planar gold substrate in a scanning tunneling microscope (STM).[^4c^, ^4g^] The probe is insulated to expose only the tip apex (see Methods in Supporting Information), the potential of the substrate is controlled at a voltage of 0.2 V, and the initial current is pre‐set to 10 pA between the electrodes. In conceptual contrast to our first recognition study,^[4c]^ both electrodes, and not only one, are functionalized with CB7 as the recognition reagent. This double functionalization expands the scope of accessible analytes widely, because a deep immersion of a suitably sized substrate into a single host cavity is no longer required.

**Figure 1:**
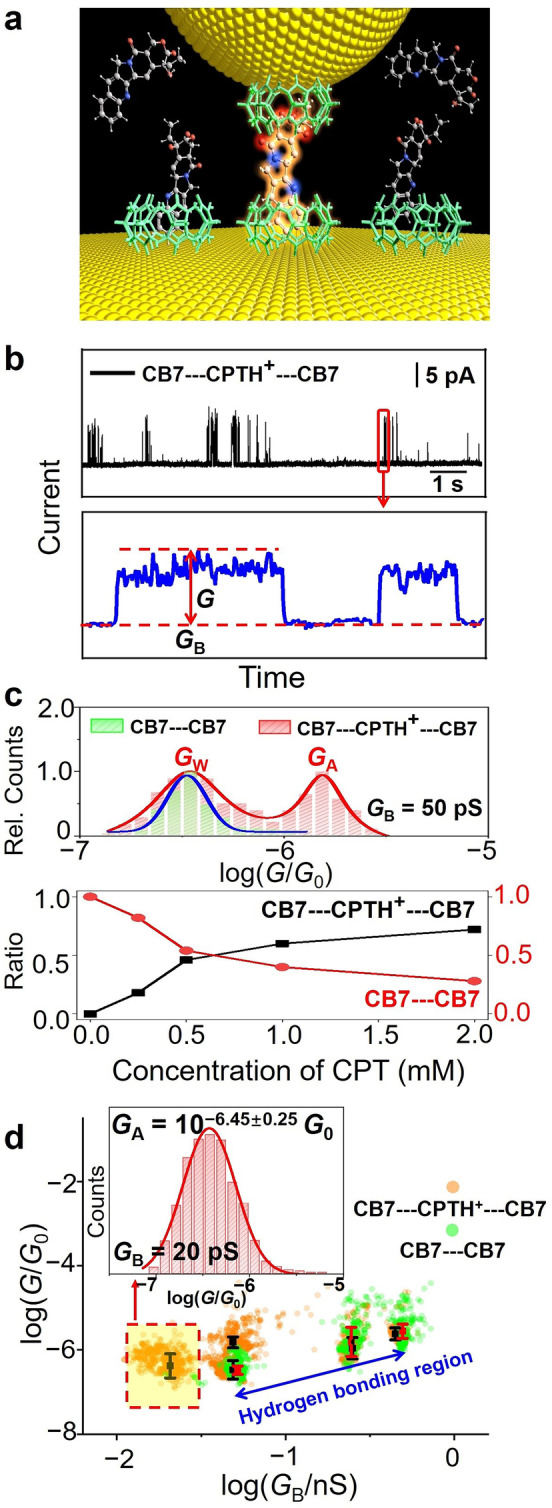
Recognition tunneling measurements. a) Schematic diagram of single‐molecule electronic recognition of CPT with CB7 (see Scheme 1) as a reader molecule attached to both electrodes. b) Typical current‐time traces recorded in water at pH 2 with CPT present at the electrode gap (*G*_B_=20 pS). The lower panel shows the zoomed‐in plot of the circled region in the upper plot. c) The conductance distribution for molecular CB7‐CB7 junctions with (red) and without CPT (green) measured at *G*_B_=50 pS (upper plot). The lower plot shows the statistical event frequency of the corresponding two conductance states recorded at different concentrations of CPT. d) Logarithmic display of the scatter plots of *G* versus *G*_B_ for molecular CB7‐CB7 junctions with (orange) and without CPT (green). The inset shows the conductance histogram of a CB7‐CPTH^+^‐CB7 junction at *G*_B_=20 pS.

In aqueous solution, the associated p*K*_a_ values of uncomplexed CPT lie at 1.2 and ≈7.^[7]^ Different states of CPT, including the protonated lactone, neutral lactone, and the ring‐opened carboxylate forms (see Scheme 1), predominate at different pH. Our first set of measurements refers to pH 2, where CPT is quantitatively protonated in its CB7 complex (CPTH^+^), owing to a complexation‐induced p*K*_a_ shift.[^1c^, ^8^] When 0.5 mM CPT is added at pH 2, distinctive and frequent switching signals are detected in the current‐time traces as shown in Figure 1b. Two parameters, *G*_B_ and *G* can be obtained from each switching signal as described previously.[^4c^, ^9^] *G*_B_ is the baseline conductance (*G*_B_=*I*_B_/*V*, here *G*_B_=20 pS), which is inversely exponentially related to the electrode gap distance, and *G* is the amplitude of the switching conductance above *G*_B_, representing the electronic conductance of a single molecule transiently bridging both CB7‐functionalized electrodes.^[10]^

In the absence of CPT, similar signals can be observed, but their frequency is significantly reduced and a large variation in *G* distribution is observed, as shown in the upper plot of Figure 1c. Two distinctive conductance peaks are obtained for CPT‐loaded junction measurements (red columns); one at a lower conductivity, near *G*_W_, that overlaps with the conductance distribution without CPT, and one at a higher conductivity, near *G*_A_, that is only observed in the presence of CPT. We realized that the experiment reported dynamically and quantitatively on the distribution between free host and CPT‐complexed host, and could be used to characterize a reversible host–guest binding phenomenon. Indeed, titration‐type experiments by increasing the CPT concentration from 0.25 to 2 mM showed increasing event frequency for the *G*_A_ signals and a decreasing rate for G_W_ (see lower panel of Figure 1c and histograms in Supporting Information). From the integrals, the binding affinity of CPTH^+^ (as guest) to the junction (as host) was estimated as ca. 1000 M^−1^. This establishes a conductance host–guest titration with rapid reversibility of binding of the analyte at a supramolecular host–guest–host junction.

As can be seen from the measured absolute conductance values, the presence of CPT in the tunneling gap (equivalent to the formation of a 2 : 1 host–guest complex) enhances the system conductance by one order of magnitude (see fittings to the two peaks). Spectroscopic studies by UV/Vis, fluorescence, and NMR of the host–guest complexes between CPT and CB7 show that CPT is partially encapsulated inside the CB7 cavity and that CPT can also form a 2 : 1 host–guest complexes with CB7 which are reminiscent of the CB7‐CPTH^+^‐CB7 junction.[^8^, ^11^] When only one electrode is functionalized with CB7, no comparable signal is observed (Figure S1), which establishes both, the stoichiometric pattern of the junction and the substrate‐scope advancement compared to our initial single‐labeling study. Due to the aromatic nature of CPT, a higher conductance (than that of CB7‐CB7 or that of water) is sensible and experimentally observed. Additional experiments were carried out at different gap distances with *G*_B_ ranging from 20 to 500 pS. Scatter plots of *G* versus *G*_B_ for both CPT‐loaded and empty junctions are summarized in Figure 1d. At *G*_B_ values of 200 and 500 pS, there is not much difference in *G* between the two systems, suggesting that the conductance signals are mainly contributed by CB7 pairs. However, when *G*_B_ is reduced to 20 pS (≈2.2 nm gap), the switching signals become rare in the blank control experiments, suggesting that the gap distance is too large in this case for conductance through the CB7‐CB7 junction to occur, while the distance is still sufficiently close to allow a CPT molecule to bridge two CB7 molecules through a conductive CB7‐CPTH^+^‐CB7 junction, as shown in the scatter points noted with a square. Histogram analysis of these enclosed data points yields a log(*G*_A_/*G*_0_) value of −6.45±0.25 (see inset of Figure 1d). The value of *G*_A_ at a particular *G*_B_ where no conductivity through CB7‐CB7 junctions is observed can be defined as a specific single‐molecule conductance of a particular analyte or state, e.g., of the protonated lactone form of CPT (dominant at pH 2).

Next, we performed pH titration experiments to monitor the conversion of different CPT states at *G*_B_=20 pS (the gap distance where no background from the vacant junction interfered). Typical current‐time traces (see Supporting Information) showed clear switching signals below pH 8, but the signals are rare when the pH value exceeds 8. This is likely due to the negative charge in the carboxylate form of CPT, which greatly reduces the affinity to the CB7 macrocycle, a known receptor for neutral and cationic guests. We verified the negligible binding of the carboxylate form of CPT (at pH 12) to CB7 by performing UV/Vis, fluorescence, and NMR titrations (see Figure S6). To quantitatively analyze the signal features observed at pH 2 to 8, we compiled the switching signals and constructed a series of two‐dimensional (2D) current‐time histograms at each pH (see Figure S4). Gaussian fittings to the *G*_A_ histograms obtained at different pH (Figure S5) show declined counts of signals with increasing pH, together with a shifted *G*_A_ position, as summarized in Figure 2a. The drop in signal frequency can be explained through the pH‐dependent conversion of CPT from the protonated to the neutral form, where the latter has a lower binding affinity to CB7, resulting in a smaller degree of complexation. It transpires that supramolecular binding equilibria in aqueous solution as well as the protonation states of the involved binding partners can be gauged by single‐molecule electrical measurements via dynamic signal frequency changes.

**Figure 2:**
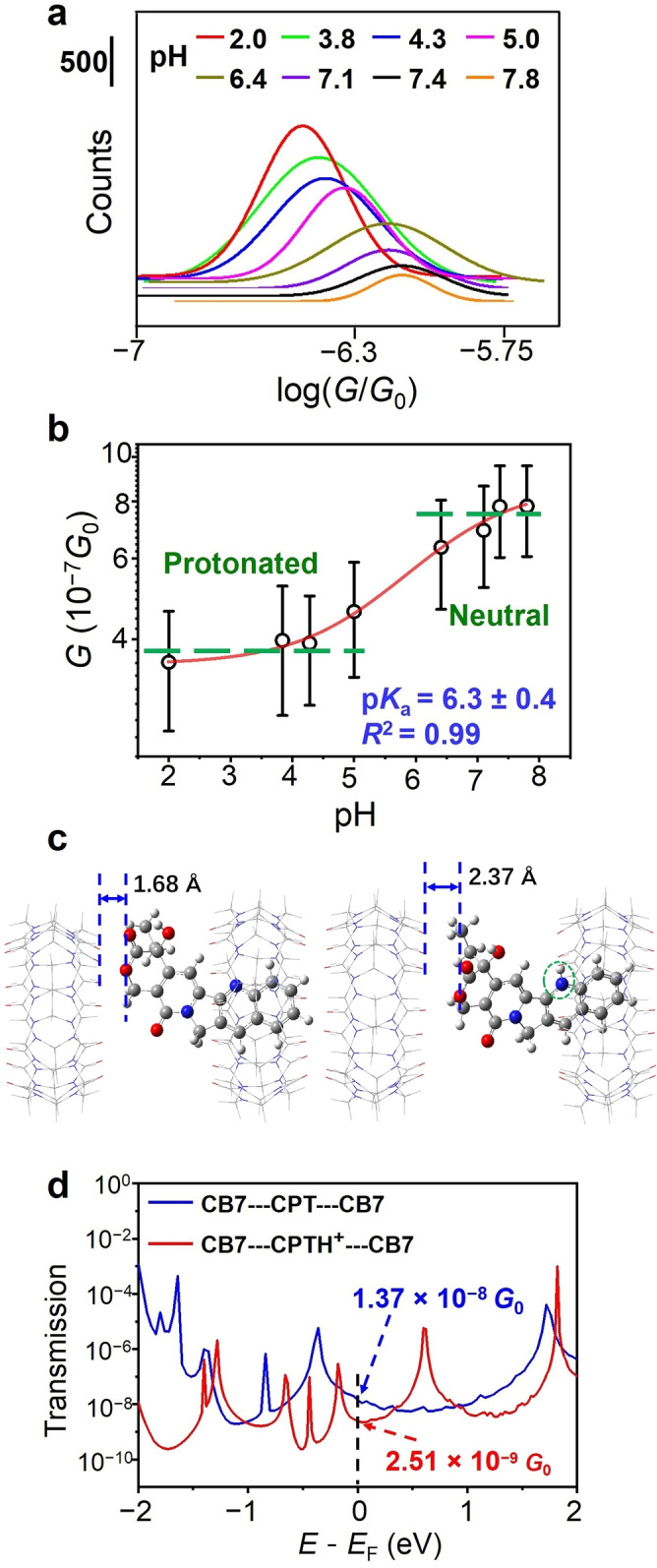
a) Gaussian fittings to the conductance profiles obtained at different pH values show a reduced peak amplitude and a shifted peak position with increasing pH. b) The conductance distribution of the CB7‐CPT‐CB7 junctions at different pH; error bars are full widths at half maximum of the conductance peaks. c) DFT‐D3 optimized structures of the isolated supramolecular junctions (2 : 1 complexes) between neutral CPT (left) or protonated CPT (right) and CB7. d) Calculated transmission functions for the neutral CB7‐CPT‐CB7 (blue line) and protonated CB7‐CPTH^+^‐CB7 junction (red line).

The shift of the *G*_A_ positions is indicative of the pH effect on the single‐molecule conductance of CPT in these supramolecular junctions. Figure 2b summarizes the statistical conductance of CPT in dependence on pH, affording a sigmoidal functional relationship between the molecular conductance and pH, which is consistent with the postulated two‐state protonation equilibrium. While pH effects on single‐molecule conductance signals are documented for cysteine‐containing peptides or thiol‐labeled azulenes,^[12]^ our technique allows the investigation of unlabeled, noncovalently bound, and therefore reversibly exchangeable analytes; in this manner, the affinity of the analyte to the junction can be modulated through a pH stimulus (see below). From the titration in Figure 2b, a p*K*_a_ value of 6.3±0.4 can be derived for CPT in the CB7‐CPTH^+^‐CB7 junction. This value is in excellent agreement with the p*K*_a_ value reported by Hazra and co‐workers for the CB7⋅CPTH^+^ complex in homogeneous solution.^[8]^ It demonstrates that the supramolecular method is not only suitable for the detection of different states of drug molecules, but that it can additionally be used to characterize the complexed analytes through their characteristic p*K*_a_ values. In addition, it is also possible to extrapolate the characteristic log(*G*_A_/*G*_0_) value of the neutral CPT as −6.10±0.04, which is significantly higher than that of the protonated CPTH^+^ form (−6.45±0.25, see Figure 2b).

To rationalize the higher conductance of the neutral (CB7‐CPT‐CB7) than the protonated (CB7‐CPTH^+^‐CB7) junctions, we carried out geometry optimizations of the two gold‐bound states by using dispersion‐corrected DFT‐D3 calculations at the B3LYP/6‐31g(d,p) level.^[13]^ The same approach has been employed to study the electronic structures of isolated host–guest^[14]^ and other complexed molecular systems.^[15]^ Figure 2c shows the optimized configurations of the isolated neutral and protonated CPT junctions, that is, of the 2 : 1 host−CPT complexes. In both modeled junctions, the quinoline moiety of CPT inserts into one CB7 cavity, driven by a combination of electrostatic and dispersion interactions. However, the penetration depth differs distinctly, because the neutral quinoline ring is completely immersed, while the protonated quinoline ring retains ion‐dipole interactions with the carbonyl portal of CB7 (dashed green circle in Figure 2c). These additional interactions strengthen the complex (higher affinity, see above) but prevent an equally deep inclusion. The resulting configuration yields a greater transmission magnitude for the neutral junction compared to the protonated one (see Figure S9) and the absolute value at the Fermi energy level is characteristically larger for the neutral CPT junction (Figure 2d), as experimentally observed. The computed conductivities cannot be compared in absolute terms (e.g., due to the neglect of solvent‐assisted conductance), but both, experiment and theory, suggest the difference in conductance to be less than one order of magnitude. It is gratifying to see that this small variation is sufficiently large to become detectable in our experimental tunneling set‐up.

Another asset of supramolecular host–guest systems is that they respond dynamically to external stimuli. In the simplest case, the addition of a competitor or even common salts leads to the displacement of the guest. Accordingly, we explored the dynamic release process of a single CPT molecule in our tunneling platform, through a gradual change in conductance behavior with increasing Na^+^ concentration. Figure 3a shows a time‐series measurement of recognition tunneling signals at the CB7‐functionalized electrode gap at the optimal *G*_B_ value of 20 pS. For the first 20 minutes in de‐ionized water, no recognition tunneling events were observed, and the typical current‐time trace shows a clean background (bottom trace in Figure 3a). Once CPT is added at a concentration of 0.5 mM, dramatic jumps occur in the current. We statistically analyzed the data sets recorded every five minutes and found that the frequency of binding events increased at the beginning and reached a plateau after ≈10 min, indicative of a host–guest binding equilibrium. When 1 M NaCl was subsequently added, the occurrence frequency of recognition tunneling signals dropped immediately and became minor after ca. 30 minutes. Repeated release and binding studies of CPT in the supramolecular junction were also carried out successfully (Figure S18). The distinctive current spikes and the time‐resolved signal frequency changes were clear indications of the real‐time detection and monitoring of a single CPT molecule being reversibly trapped and released from the CB7‐functionalized platform. Moreover, the conductivity response is continuous, as indicated by the constant variation towards a saturation level, which could eventually allow for continuous flow screening set‐ups for different analytes. These features, along with the added possibility to detect analytes in different protonation or hydration states, renders it an analytical technique in spe. As an equivalent to known supramolecular phenomena, this experiment constitutes a detection by a competitive conductance titration, where the salt displaces the indicator at the tunneling gap.

**Figure 3:**
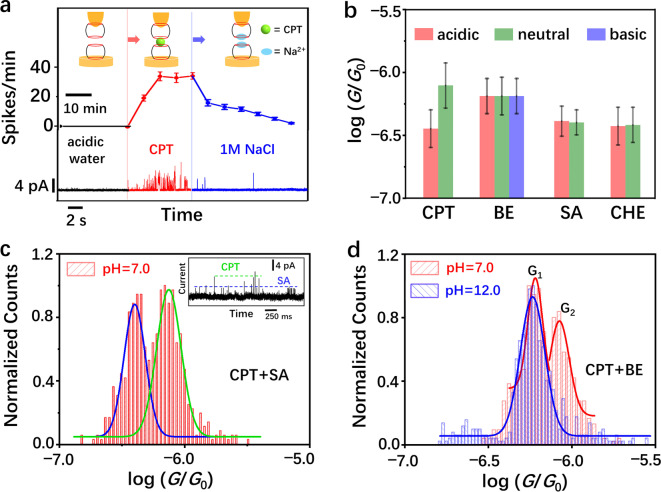
a) Time‐series recognition tunneling measurement of a single CPT molecule first captured in a supramolecular junction and subsequently released upon addition of cations. Typical time‐series traces are displayed at the bottom. The black line shows the current response of the system in acidic water (pH 2, *G*_B_=20 pS), the red line shows the temporal evolution of conductance upon addition of CPT (0.5 mM), corresponding to the formation of a CB7‐CPTH^+^‐CB7 junction, and the blue line shows the continuous reversal of the effect upon addition of Na^+^ ions (1 M NaCl), which displaces CPT from the junction. Shown in the middle is a plot of occurrence frequency versus time for switching spikes arising from the CB7‐CPTH^+^‐CB7 junction. b) Conductance distributions for different drug molecules (CPT, BE, SA, and CHE) in acidic (pH 2), neutral (pH 8 for CPT and pH 7 for the rest), and basic (pH 12) solutions; note that only BE afforded a detectable conductance in basic condition. c) Conductance distribution of the recognition tunneling measurement in a mixture of CPT and SA (both 0.5 mM) at pH 7. Two distinctive peaks are generated and the peak values are consistent with the results of the single‐molecule conductance of CPT and SA measured separately (Figure 3b). The inset displays a typical current‐time trace showing distinctive current spikes. d) Conductance histograms of the recognition tunneling events measured in a mixture of CPT and BE (both 0.5 mM) at pH 7 and 12. Two distinctive peaks are shown at pH 7, while only one peak, with the peak value corresponding to BE, is shown at pH 12.

Finally, we performed a mini‐screening of related drug molecules to testify the versatility of our technique (Scheme 1). SA and CHE were studied because they are also known to change their state and bioactivity in dependence on pH, with a conversion from a potent positive (iminium) to an inactive neutral (alkanolamine or pseudobase) form. As a pH‐*insensitive* drug molecule, we selected BE,^[6]^ which would serve as a “negative control”, the results are summarized in Figure 3b and Table S3. As expected, the single molecule conductance of BE remained unchanged, within error, across the wide pH range, with a log(*G*_A_/*G*_0_) value of −6.19±0.14 at pH 7. The recognition tunneling signals of SA and CHE are detectable at pH 2 and 7, but vanished at pH 12, suggesting that only their positively charged iminium states (those depicted in Figure 3b) were able to bind to the CB7 junction. This result is consistent with the molecular‐recognition properties of CB7, which preferentially binds cations. Further experiments on binary mixtures of the drug molecules showed that CPT can be differentiated from SA or BE via their conductance bands (Figure 3c and d). In addition, a pH variation from 7 to 12 eliminated the conductance peak for CPT, allowing selective recognition of BE (Figure 3d). These findings expose the advantages of the supramolecular junctions, because chemically unmodified analytes can reversibly and competitively produce characteristic single‐molecule conductance signals that additionally respond predictably to external stimuli such as pH and salts.

In conclusion, we have shown that a series of biologically relevant analytes can be reliably characterized in CB7‐modified tunneling junctions. By setting up two CB7‐decorated electrodes at a fixed‐gap distance, the reversible capture of target molecules can be monitored in real time via the appearance of distinct recognition tunneling signals. The molecular states of CPT at the CB7 junction, which can be discriminated by the frequency of recognition tunneling signals, change systematically and dynamically upon variations of the CPT concentration (complexed/uncomplexed equilibrium, minute time scale) and of the pH (protonated/unprotonated equilibrium, immediate response). Moreover, we show that an external stimulus of salt (Na^+^, Ca^2+^) erases the recognition tunneling signals of CPT by competitive binding, indicating controlled binding and release. Importantly, the reliable detection of such large, anchor‐free single molecules provides a complementary strategy for single‐molecule detection and electronics.

## Conflict of interest

The authors declare no conflict of interest.

## Supporting information

As a service to our authors and readers, this journal provides supporting information supplied by the authors. Such materials are peer reviewed and may be re‐organized for online delivery, but are not copy‐edited or typeset. Technical support issues arising from supporting information (other than missing files) should be addressed to the authors.

Supporting Information

## Data Availability

The data that support the findings of this study are available in the Supporting Information of this article.
