# Supplementary material for: Dynamic Interconversions of Single Molecules Probed by Recognition Tunneling at Cucurbit[7]uril‐Functionalized Supramolecular Junctions
Source: Angew Chem Int Ed Engl. 2022 May 5;61(26):e202203830. doi: 10.1002/anie.202203830 (PMC9324061; doi:10.1002/anie.202203830)
Supplement: Supplementary file 1 — Supporting Information [file ANIE-61-0-s001.pdf]

Angewandte Chemie
Eine Zeitschrift der Gesellschaft Deutscher Chemiker

Supporting Information

Dynamic Interconversions of Single Molecules Probed by Recognition Tunneling at Cucurbit[7]uril-Functionalized Supramolecular Junctions

B. Xiao, S. He, M. Sun, J. Zhou, Z. Wang, Y. Li, S. Liu, W. M. Nau*, S. Chang**

Table of Contents

S1 Recognition tunneling method

S2 Conductance measurements in dependence on CPT concentration

S3 Typical tunneling traces recorded at different pH values

S4 Two-dimensional histograms of CB7---CPT---CB7 junctions at different pH values

S5 Conductance distribution of CB7---CPT---CB7 junctions at different pH values

S6 Binding of CB7 to camptothecin

S7 DFT calculations for CB7---CPT---CB7 junctions

S8 Binding of CB7 to sanguinarine

S9 Binding of CB7 to chelerythrine

S10 Repeated release and binding studies of CPT at the supramolecular junctions

S11 Summary of analyte affinities to CB7 and their molecular conductance values at the supramolecular junctions

S12 Cartesian coordinates of the isolated CPT structures and the CB7---CPT junctions obtained by the DFT calculations

S1. Recognition tunneling method

Preparation of test solutions. Cucurbit[7]uril (CB7) was prepared according to the reported procedure[1]. Camptothecin (CPT), sanguinarine (SA), chelerythrine (CHE), and berberine (BE) were purchased from Sigma Aldrich and used without further purification. Owing to the moderate solubility of CPT, SA and CHE, stock solutions of 5 mM were prepared in DMSO in order to allow the titration experiments. 0.5 mL of the CPT stock solution was added to Milli-Q water at the desired pH (adjusted with HCl_{aq} and $NaOH_{aq}$) and the solution was subsequently sonicated for 10 min to ensure rapid mixing. Test solutions of the other analytes were prepared accordingly.

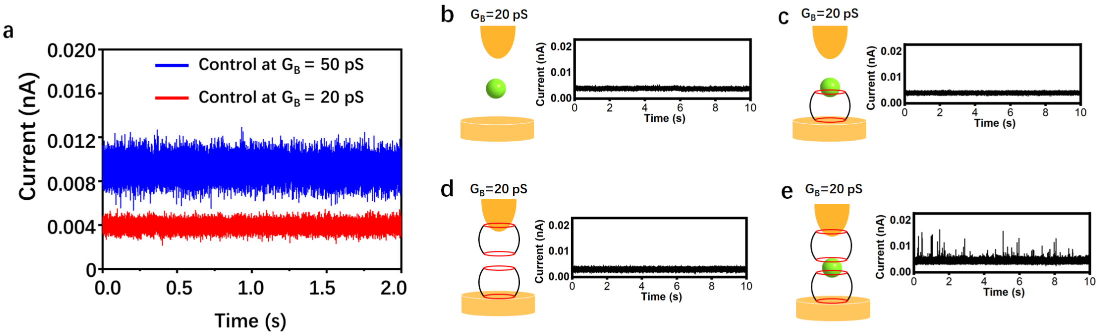

Figure S1. (a). Control current traces at G_B = 50 pS and 20 pS showing a clean background and low electronic noise. b-d. Negative control experiments (without current switching signals) carried out under different experimental conditions, namely (b) with both electrodes non-functionalized, (c) a single electrode functionalized, and (d) without target molecules. (e) Typical recognition tunneling measurement with both electrodes functionalized and the target molecules (CPT, as an example) added. Distinctive current spikes are frequently observed in the current-time traces.

Tunneling measurements. The preparation and chemical modification of the STM tip and the substrate has been described in our previous work.[2] The conductance measurements were performed at ambient temperature on a Keysight 6500 instrument. Before the conductance experiment, the distance between STM tip and substrate was adjusted to maintain a gap of set-point = 4 pA and the instrument was warmed up for 2 hours to stabilize the tip. All tunneling traces were collected at a bias of 0.2 V with a sampling rate of 10 kHz. A blank control experiment (without CB7 reader molecules) was performed with the HDPE-coated tip for a leakage test. The low-noise level in the tunneling traces at G_B = 50 pS (Noise = 2 pA) and 20 pS (Noise = 1 pA) indicated that the insulation probe is capable of single-molecule electronic detection (Figure S1a). In addition, we carried out control experiments in the STM studies, as shown in Figure S1 b-d. No current switching signals are generated when the electrodes are not functionalized (b) or only one electrode is functionalized (c), or when no target molecules are present (d). The current jump signals only appear when both electrodes are functionalized with CB7 and the target molecule is present in solution. Statistical analysis of the current spikes was automated by using home-built Labview programs as described

previously[2]. The conductance histograms comprised current spikes extracted from thousands of individual $I(t)$ traces and the error bars were calculated from the full width at half-maximum (FWHM) of the fitting peaks.

S2. Conductance measurements in dependence on CPT concentration

As shown in Figure S2, the conductance peaks at G_B = 50 pS remain unchanged, while the proportion of molecular junction events in G_A (CB7---CPTH$^+$---CB7) and G_W (CB7---CB7) increases with increasing concentration of CPT. The area of signal counts of G_A and G_W are integrated respectively and compared, the ratios of the two areas are listed in Table S1.

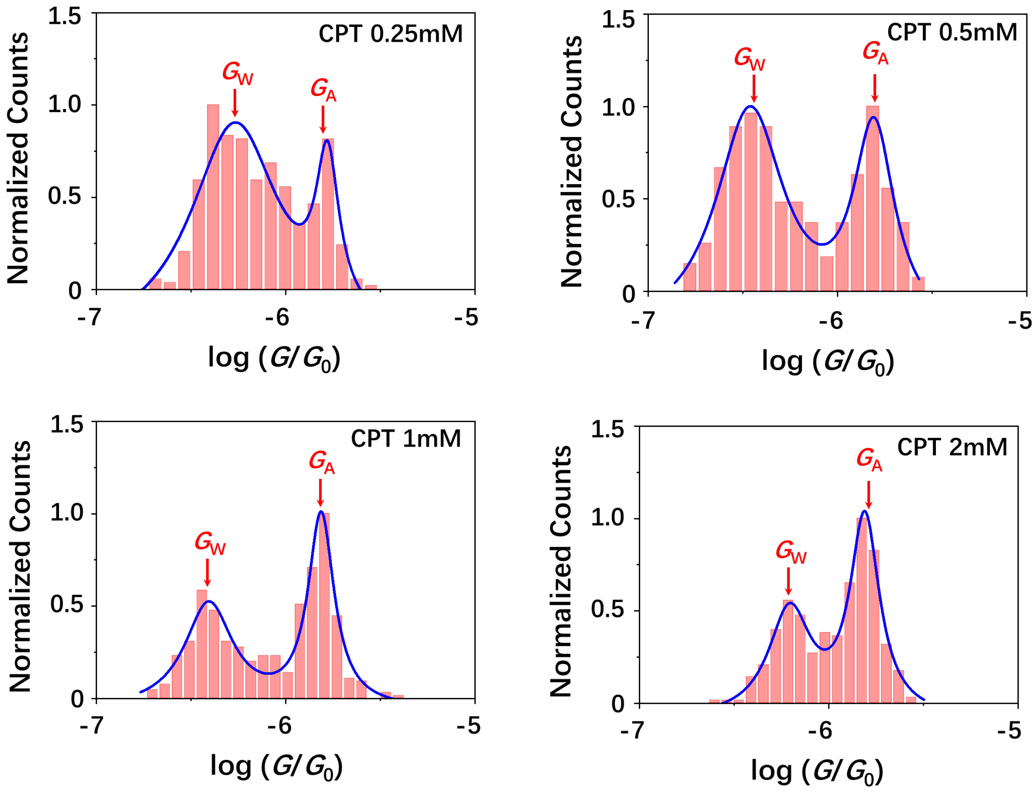

Figure S2. Signal distributions of G_W and G_A measured at G_B = 50 pS in acidic solution (pH 2) with concentration of CPT varied from 0.25 mM to 2 mM.

Table S1. Conductance parameters of junction events in G_w and G_A measured at G_B = 50 pS in acidic solution (pH 2) with different CPT concentrations.

C_{CPT} (mM)	Log(G/G_0)		Area ratio	
	G_W	G_A	G_W	G_A
0.25	−6.26 ± 0.29	−5.77 ± 0.07	0.82	0.18
0.5	−6.46 ± 0.25	−5.80 ± 0.15	0.54	0.46
1	−6.40 ± 0.16	−5.81 ± 0.08	0.40	0.60
2	−6.21 ± 0.14	−5.81 ± 0.10	0.30	0.70

S3. Typical tunneling traces recorded at different pH values

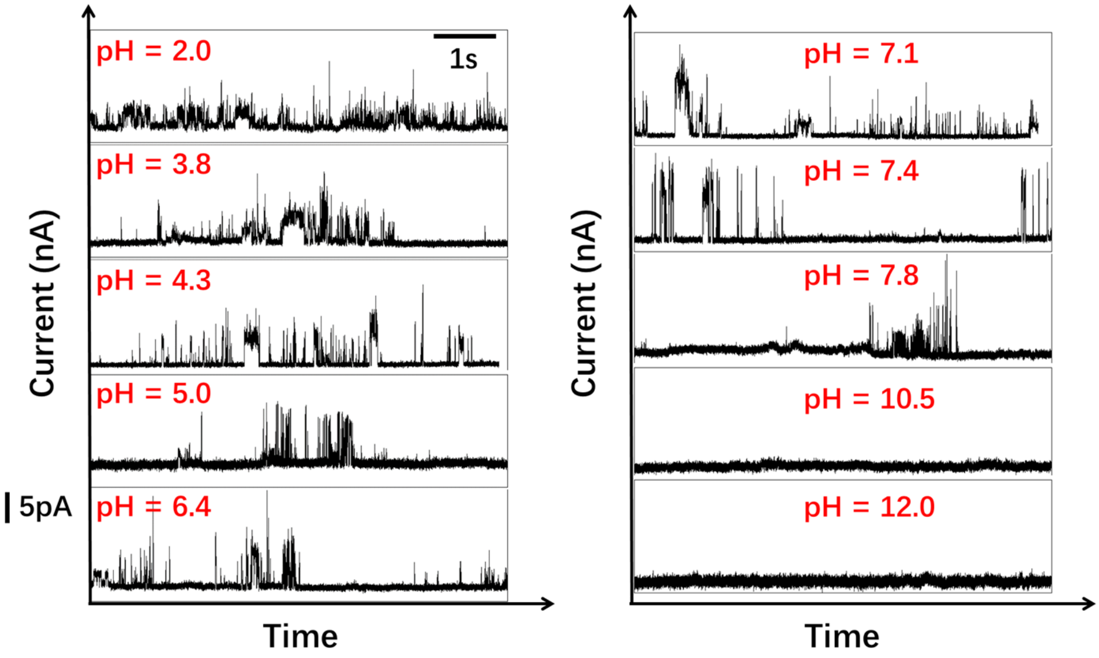

Figure S3. Typical current-time traces of CB7---CPT---CB7 junctions obtained from pH 2 to pH 12.

S4. Two-dimensional histograms of CB7---CPT---CB7 junctions at different pH values

The 2D histograms were constructed by overlaying the selected current spikes from hundreds of individual *I*(*t*) traces in each experiment over a wide pH range, using bins of 5 ms along the time axis and $10^{0.05}$ G_0 bins along the conductance axis. We plotted the 2D histogram by setting the origin of the time axis to the start point at which the current suddenly increased and the end until it came back to the set-point current. As can be seen in Figure S4, the counts of constructed histograms decrease with increasing pH value, indicating that the CB7---CPT---CB7 supramolecular junction depletes at high pH. As the solution became less acidic from pH 2 to pH 6, the initially dominant protonated lactone form of CPT gradually converted into the neutral lactone form which bound less strongly to the junction compared to the protonated form. Besides, the proportion of CPT carboxylate form (which interacted only to a negligible extent with CB7) increased concomitantly above pH 6, as observed from the UV-vis absorption and fluorescence spectra (see Section S6), which further minimized the formation probability of the supramolecular junctions. In contrast to the reduction of the events probability, the absolute conductance values increased gradually from pH 2.0 to 7.8, as marked by the blue lines in Figure S4 a-h.

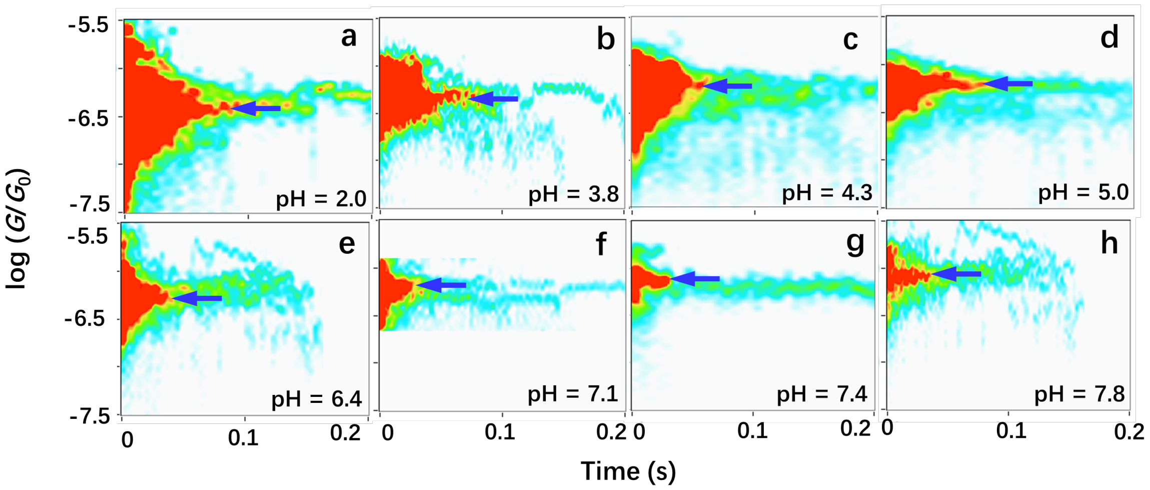

Figure S4. Two-dimensional histograms of CB7---CPT---CB7 junction conductance in aqueous solution of pH 2.0 (a), 3.8 (b), 4.3 (c), 5.0 (d), 6.4 (e), 7.1 (f), 7.4 (g), 7.8 (h). The bin size was 5 ms for the time and $10^{0.05}$ G_0 for the conductance.

S5. Conductance distribution of CB7---CPT---CB7 junctions at different pH values

The conductance values at different pH were extracted from the 2D histogram and further constructed 1D conductance histograms (logarithmic bin 200 bins along the conductance axis). Overlaid 1D conductance histograms for CB7---CPT---CB7 junction over pH range of 2.0-7.8 are shown in Figure S5. The conductance histograms suggest that the conductance increases slowly from pH 2.0 to 4.3 and rises up rapidly from pH 6.4 to 7.8.

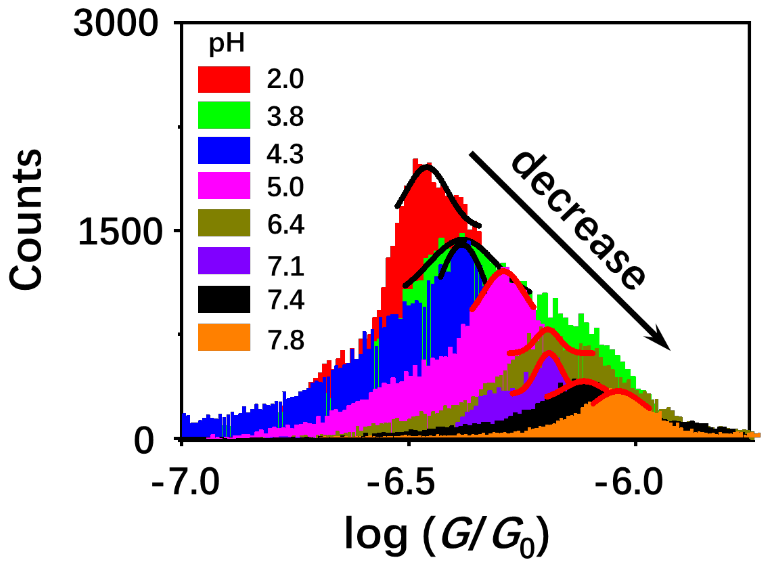

Figure S5. pH-dependent conductance distribution histograms and the corresponding Gaussian fittings for the CB7---CPT---CB7 peaks at each pH value.

Table S2. CB7---CPT---CB7 junction conductance parameters at different pH values

pH	Conductance (× 10^{-7} G_0)	Peak width (× 10^{-7} G_0)
2.0	3.56	1.04
3.8	3.98	1.25
4.3	3.93	1.05
5.0	4.59	1.29
6.4	6.35	1.70
7.1	6.91	1.72
7.4	7.79	1.76
7.8	7.81	1.75

S6. Binding of CB7 to camptothecin

Previous studies on CPT revealed its structural changes with the pH of the solution, which largely increases the complexity of the CB7•CPT binding system. Douhal *et al.* reported a pK_a value of 1.85 for the excited-state of CPT.[3] Meanwhile, Hazra *et al.* obtained a pK_a value of 1.18 for CPT in the ground-state; they also found that a large excess of CB7 in solution can shift the pK_a of CPT to 6.8 and 6.2, respectively, at the excited and the ground state.[4] It was assumed that by forming a 1:2 complex with CB7, the protonation of the quinoline nitrogen became easier, possibly because the electron density is enriched by trapping between the portals of two CB7 hosts.[4] As the pH rises, the carboxylate form of CPT gradually takes over the lactone form, and, as a result, the pharmacological activity of CPT is diminished. Above pH 8, CPT exists mostly in its carboxylate form.[5] Dong *et al.* studied the binding behavior of CPT and CB7 at pH 2,[6] and confirmed the formation of a 1:2 complex, as had been observed by Hazra near neutral pH. However, experimental structural information on the 1:2 complex remains elusive. Nonetheless, the 1:1 complex prevails in solution if CB7 and CPT are employed in low concentrations. This allowed us to use a 1:1 binding model in the fitting, the obtained binding constant is 8.4 × 10^5 M^{-1} and 7.9 × 10^5 M^{-1}, respectively, from UV-vis and fluorescence titration. The increasing absorption at 407 nm as well as emission at 515 nm refers to the growing fraction of the protonated form, which is induced by encapsulation within CB7 (Figure S6). ^{1}H NMR studies of CPT and CB7 in D_2O revealed an upfield shift of the quinoline ring protons, while the protons on the other rings shifted downfield, indicating a preferential immersion of the quinoline ring inside the CB7 cavity while the other part of the drug molecule remains positioned outside the cavity[6]. This ^{1}H NMR shift pattern corresponds better to a partial 1:1 inclusion complex than to a 2:1 inclusion complex, such that the second CB7 macrocycle interacts likely very weakly, and more superficially with CPT. At basic pH

values, we found no shift of the ^{1}H NMR peaks of CPT upon addition of 2 equiv. of CB7, suggesting the absence of detectable binding; this is in line with the spectroscopic titration results (see Figures S6 and S7). We were not able to measure the ^{1}H NMR in acidic D_2O, because the solubility of CPT was too low in this medium.

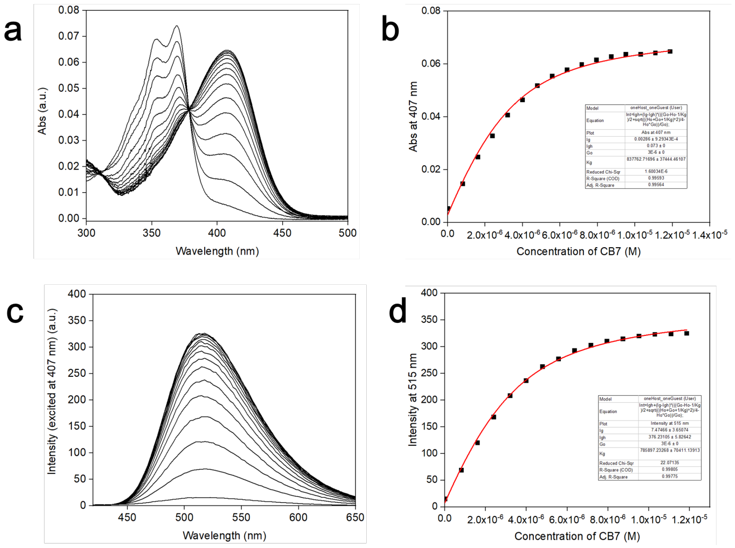

Figure S6. UV-vis and fluorescence spectra of CPT at pH 2. a. UV-vis spectral changes caused by gradual addition of up to 12 µM CB7 into a 3 µM CPT solution at pH 2. **b.** The absorbance at 407 nm was plotted against the concentration of CB7 and a binding constant of $(8.4 \pm 0.4) \times 10^{5}$ M^{-1} was obtained by fitting according to the 1:1 binding model. **c.** Fluorescence spectra of 3 µM CPT in acidic solution (pH 2) upon addition of up to 12 µM of CB7 (excited at 407 nm). **d.** The emission intensity at 515 nm was plotted against the concentration of CB7 and a binding constant of $(7.9 \pm 0.7) \times 10^{5}$ M^{-1} was obtained by fitting according to the 1:1 binding model.

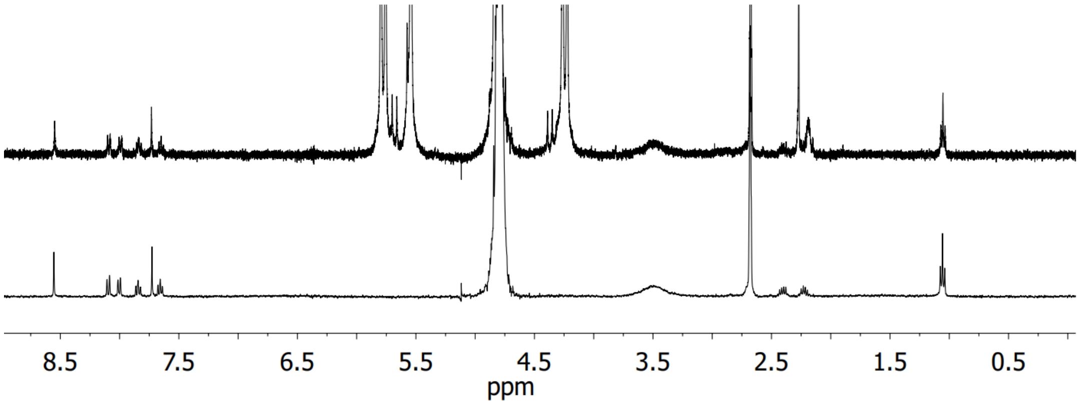

Figure S7. ^{1}H NMR spectra of 0.5 mM CPT alone (bottom) and in the presence of 1 mM CB7 (top) dissolved in a solvent mixture of basic D_2O (pD 12, 90%) and DMSO-d_6 (10%). The chemical shifts of CPT remained the same upon addition of CB7, suggesting no obvious host-guest interaction.

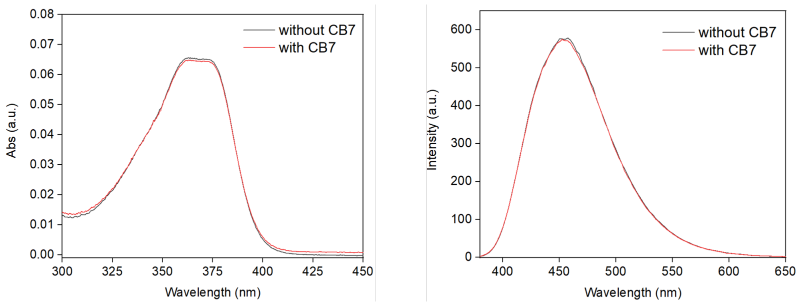

Figure S8. UV-vis absorption and fluorescence spectra (excited at 370 nm) of 3 μM CPT in basic solution (pH 12) in the absence and presence of 23.4 μM CB7. Only minor changes in the absorption and emission spectra were observed in the presence of 8 equiv. of CB7, pointing to no significant interaction.

S7. DFT calculations for CB7---CPT---CB7 junctions

Molecular geometry optimizations were carried out with the Gaussian 09 program by using dispersion-corrected density functional theory (DFT-D3) and the B3LYP functional along with the 6-31G(d,p) basis set. Grimme's D3 empirical dispersion correction with Becke-Johnson damping (GD3BJ) was used. Vibrational frequency analyses were performed for all geometry-optimized structures, confirming the absence of imaginary frequencies, and therefore identifying them as energy minima.

The host-guest complex structures were optimized between two gold electrodes to form molecular devices in the Atomistix-Tool-Kit software. We applied DFT methods within the generalized gradient approximation (GGA) by using the Perdew-Burke-Ernzerhof (PBE) exchange-correlation function to simulate the optimized structures. The single zeta polarized level was used for Au atoms and the double zeta polarized level was adopted for C, H, O, N and Cl atoms based on the linear combination of atomic orbitals (LCAO) basis set approach. The Au electrodes were settled to a 6×6×2 supercell. The isolated organic complexes were optimized and the complexes were inserted between the left and right Au electrodes, and the resulting supramolecular junctions were relaxed until the total free energy reached a minimum. During the relaxation, the force tolerance was set to 0.5 eV/Å. It is worth noting that we placed one adjacent chloride atom in the CB7---CPTH$^+$---CB7 system to simulate the effect of protonation (HCl) while maintaining an overall neutral assembly.

First principles calculations were carried out to investigate electronic transport properties. In the series of calculations, the exchange-correlation potential was approximated within the GGA-PBE functional for exchange and correlation effects. A mesh cutoff energy of 75 Hartree and a (1,1,89) *k*-point mesh within the Monkhorst-Pack scheme were utilized. The single-ζ polarization basis set was used for the gold atoms and the double-ζ basis set with polarization functions was used for all other atoms. According to the Landauer formalism,

$$G = \frac{2e^2}{h} \sum_n T_n ,$$

the conductance G of a supramolecular junction can be calculated, where e is the electron charge, h is Planck's constant, and T_n is the transmission coefficient of the individual transport channels which describes how effective a molecule performed in scattering an incoming electron from the right lead into the left lead. In this way, the conductance values of the molecule under zero bias voltage were obtained.

As shown in Figure S9, the blue and red arrows show the forward and backward electron transport pathways in the molecular system with the thickness of the arrow representing the absolute transmission magnitudes. The main transmission pathways for both systems are along Au→left CB7→CPT→Au. There is almost no arrow pointing to or from the CB7 molecule that encapsulates the quinoline ring; instead, this macrocycle serves to facilitate direct CPT---Au transmission through the formation of inclusion complexes. Therefore, the computational modelling results account for the experimental observation that the recognition tunneling signals are "switched on" upon addition of CPT, because the CPT is (expectedly, due to its aromatic character) more conductive than the aliphatic macrocyclic host. Secondly, and again in agreement with the experiment, the electron transmission modelling predicts a higher conductance for the neutral junction than for the protonated one, as indicated by the arrows connecting the right Au surface to the junction. This effect is fully consistent with the deeper immersion of the neutral quinoline ring (see Figure 2c), which places it more closely to the Au surface (3.25 versus 4.41 Å, see Figure S9). Counterintuitively, even though the neutral quinoline ring is more deeply immersed into CB7, this does not increase the distance of the lactone group to the second Au surface, but rather decreases it (10.14 and 10.64 Å for the Au-O and Au-C distances for CPT, shorter than in the case of CPTH+ with 10.71 and 10.77 Å), as a consequence of different quinoline protonation-induced tilting angles (also see Figure 2c); this shorter bridging distance for the neutral form may be a second contributor to the increased conductivity.

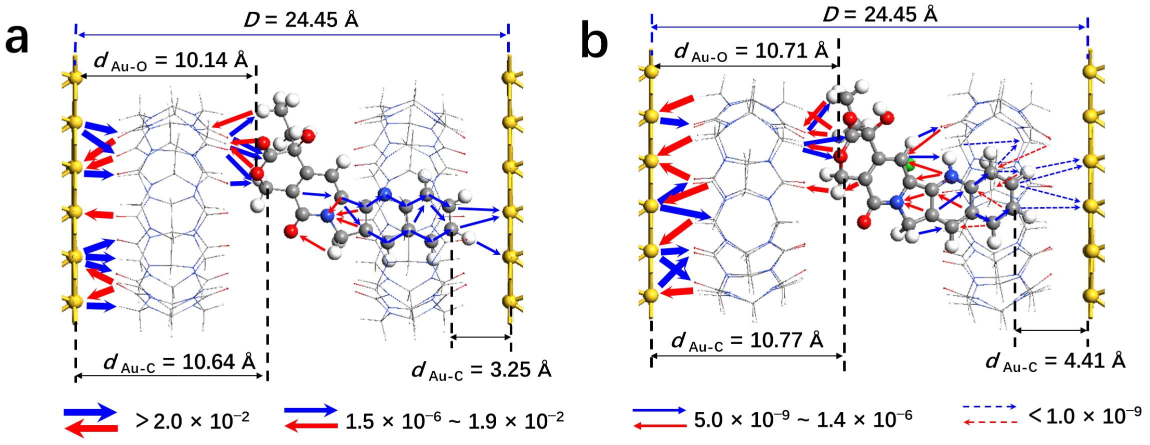

Figure S9. Electron transmission pathways in the neutral CB7---CPT---CB7 (**a**) and protonated CB7---$CPTH^+$---CB7 (**b**) junctions; blue and red arrows indicate the forward and reverse loop current, respectively, and the thickness of the arrows is proportional to local transmission contribution.

S8. Binding of CB7 to sanguinarine

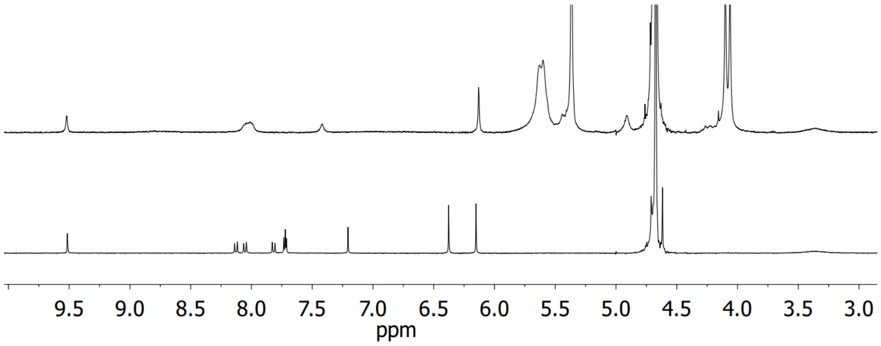

Figure S10. ^{1}H NMR spectra of 0.5 mM SA alone (bottom) and in the presence of 1 mM CB7 (top) in a solvent mixture of acidic D_2O (pD 2, 90%) and DMSO-d_6 (10%). The ^{1}H NMR spectrum in basic solution could not be recorded due to a poor solubility of the guest.

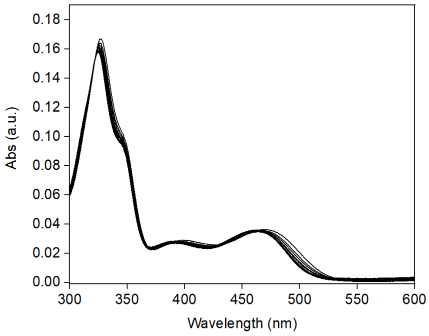

Figure S11. UV-vis absorption spectra of 6 µM SA in the absence and presence of up to 23.4 µM of CB7 in HCl solution (pH 2).

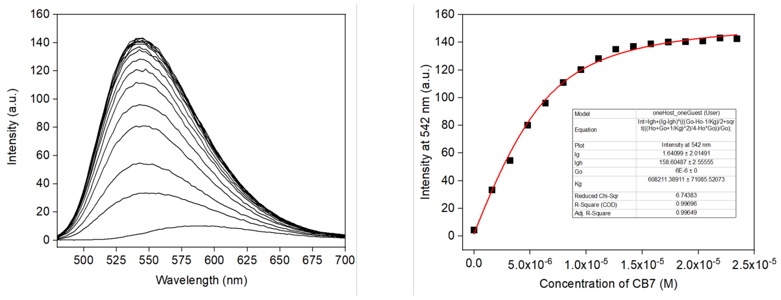

Figure S12. Fluorescence spectra of 6 µM SA in the absence and presence of up to 23.4 µM CB7 in HCl solution (pH 2, left), excited at 470 nm. The intensity at 542 nm was plotted against the concentration of CB7 (right) and a binding constant of $(6.1 \pm 0.7) \times 10^5$ M^{-1} was obtained by fitting according to the 1:1 binding model.

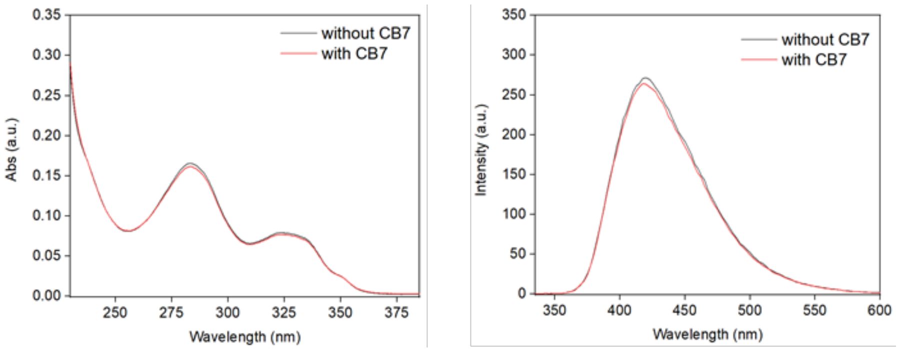

Figure S13. UV-vis absorption (left) and fluorescence spectra (right, excited at 326 nm) of 6 μM SA in basic solution (pH 12) in the absence and presence of 23.4 μM of CB7.

S9. Binding of CB7 to chelerythrine

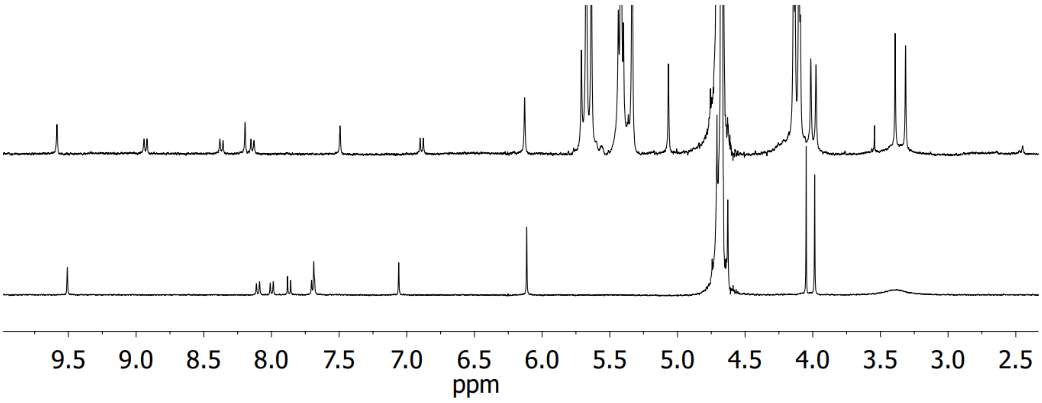

Figure S14. ^{1}H NMR spectra of 0.5 mM CHE alone (bottom) and in the presence of 1 mM CB7 (top) in DCl (pD 2). The ^{1}H NMR spectrum in basic solution could not be recorded due to a poor solubility of the guest.

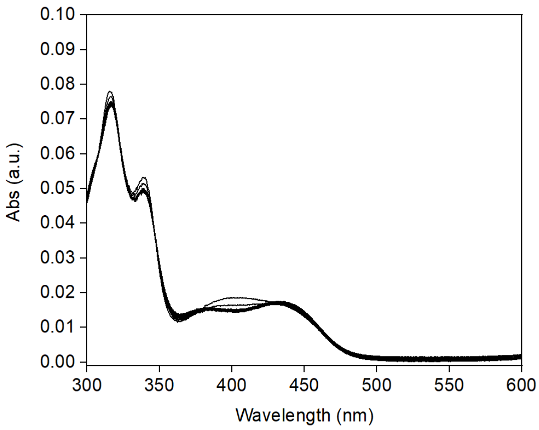

Figure S15. UV-vis absorption spectra of 3 μM CHE in the absence and presence of up to 23.4 μM of CB7 in HCl solution (pH 2).

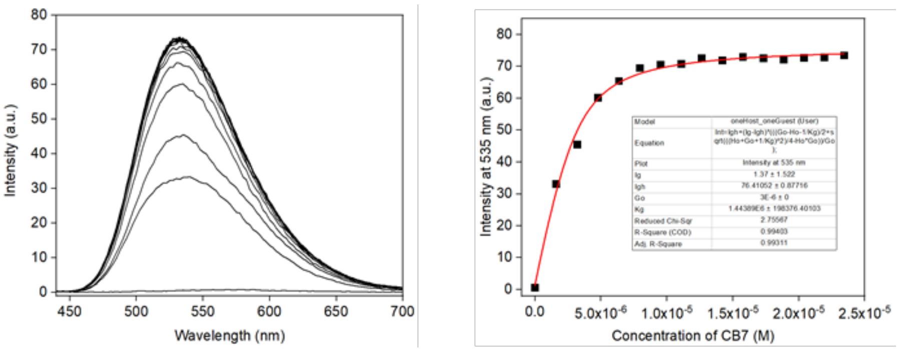

Figure S16. Fluorescence spectra of 3 μM CHE in the absence and presence of up to 23.4 μM of CB7 in HCl (pH 2, left), excited at 430 nm. The intensity at 535 nm was plotted against the concentration of CB7 (right) and a binding constant of $(1.4 \pm 0.2) \times 10^{6}\ M^{-1}$ was obtained by fitting according to the 1:1 binding model.

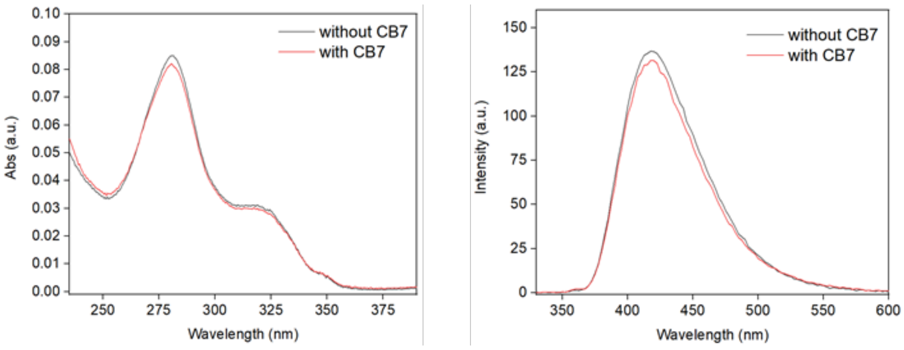

Figure S17. UV-vis absorption (left) and fluorescence spectra (excited at 319 nm, right) of 3 μM CHE in the absence and presence of 23.4 μM of CB7 in basic solution (pH 12).

S10. Repeated release and binding studies of CPT at the supramolecular junctions

We conducted repeated release/binding studies by alternatively adding CPT and salt in the STM measurement cell and monitoring the tunneling current changes, as shown in Fig. S18. The STM gap was set to an initial current of 4 pA (G_B= 20 pS) and both electrodes were functionalized with CB7. In the first 10 minutes, no current switching signals were observed in the absence of analyte. Once 0.5 mM CPT was added to the system, dramatic jumps occurred in the current and signal frequency (red points) that saturated after ca. 10 minutes. Subsequently, 0.1 M Ca^{2+} was added to the solution and an immediate reduction of the signal frequency (blue points) is seen in the time-resolved signal frequency change, suggesting the effective release of CPT from the supramolecular junction by competitive CB7 portal binding. Subsequently, we rinsed the electrodes with DI water and repeated the measurements by adding CPT and salt alternatively for another two cycles. The repeated release-binding results demonstrate the reversibility of the processes and robustness of the supramolecular junctions.

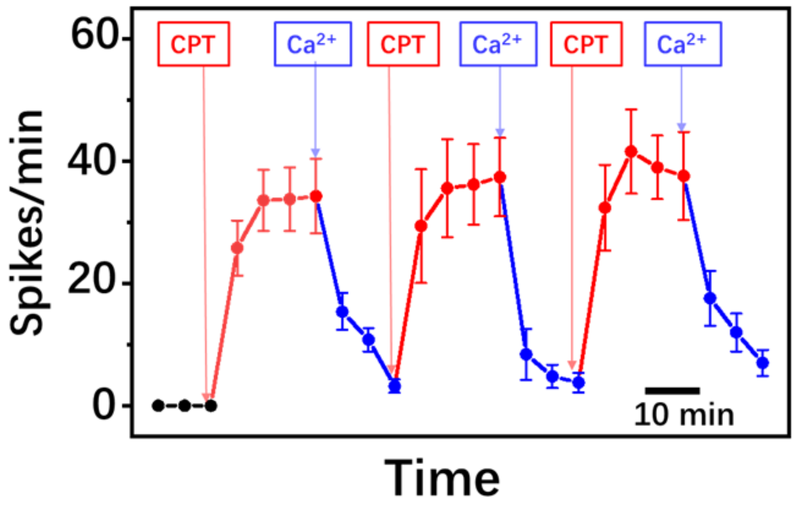

Figure S18. Repeated release and binding studies of CPT at doubly CB7-functionalized electrodes. The current jump spikes were statistically analyzed every five minutes during the repeated cycles of adding first CPT and subsequently salt. The red points represent the current signal frequency after adding CPT to the STM solution and the blue points denote the signal frequency after adding 0.1 M Ca^{2+} to the measurement solution.

S11. Summary of analyte affinities to CB7 and their molecular conductance values at the supramolecular junctions

The conductance values of all studied molecules at different pH are summarized in Table S3. The G_A values of SA and CHE are the same, within error, as might be expected from their closely related structures, but both display a slightly lower conductance than BE. In contrast to CPT, SA and CHE do not exhibit a change in conductance upon changing from acidic to neutral pH.

Table S3. Summary of the pK_a values of CPT, SA, CHE, and BE, the conductance values ($\log(G_A/G_0)$) at different pH values, and their binding constants with CB7.

Analyte	Binding constant[a] (× 10^6 M^{-1})	pK_a	$\log(G_A/G_0)$		
			pH 2.0	pH 7.0	pH 12
CPT	0.84 ± 0.04	1.2, ~7[b]	–6.45 ± 0.25	–6.10 ± 0.19[c]	n.d.[d]
SA	0.61 ± 0.07	8.3[e]	–6.39 ± 0.25	–6.40 ± 0.21	n.d.[d]
CHE	1.4 ± 0.2	9.2[e]	–6.43 ± 0.30	–6.42 ± 0.28	n.d.[d]
BE	24 ± 2[f]	-	–6.19 ± 0.14	–6.19 ± 0.15	–6.19 ± 0.14

[a] Measured by optical titrations, this work, see Supporting Information, Sections S6-S9. [b] Two pK_a values, from ref. [5,6]. [c] Measured at pH 7.8, assigned to the neutral CPT form, see text. [d] No detectable conductance signal. [e] From ref. [7]. [f] From ref.[8].

S12. Cartesian coordinates of the isolated CPT structures and the CB7---CPT junctions obtained by the DFT calculations

CB7---CPT---CB7:

O	9.99268	4.18995	0.27239
O	10.05336	2.22144	3.5298
O	10.06986	1.53407	3.99252
O	10.03625	4.22537	1.30992
O	9.96605	3.83645	2.48405
O	9.91859	0.65918	4.55673
O	9.93206	2.9175	3.32204
O	3.78681	4.68441	0.53416
O	3.80074	2.63663	3.43432
O	3.90843	1.32227	3.29065
O	3.85015	4.52354	0.96128
O	3.82732	4.3009	2.28076
O	3.78577	0.96331	3.73308
O	3.79176	2.92875	2.53916
N	8.16247	5.22997	0.6976
N	8.18947	4.95472	1.52176
N	8.20241	3.63959	3.55799
N	8.14611	1.76177	4.76377
N	8.10204	0.67194	4.89425
N	8.11397	2.78788	4.18008
N	8.16011	4.64925	2.60294
N	8.19957	5.40527	0.50039
N	8.15638	5.17989	1.91248
N	8.12923	4.09671	3.87228
N	7.96917	1.90003	4.92456
N	7.92527	0.31664	5.2421
N	7.95041	2.64194	4.50102
N	8.0229	4.19957	2.89693
N	5.70832	5.34861	0.59059
N	5.74935	5.2639	1.63932
N	5.75151	3.88257	3.64043
N	5.69634	1.8258	4.51078
N	5.66119	0.59137	4.63339
N	5.68139	2.69966	3.89244
N	5.71329	4.56776	2.35365
N	5.76387	5.7371	0.45195
N	5.73315	5.59798	1.98591
N	5.67784	4.14191	3.67934
N	8.76305	5.02791	1.60487
N	8.82648	5.47696	0.79179
N	8.82012	4.67512	2.88115
N	8.83268	2.96141	4.60215
N	8.78029	0.83885	5.30286
N	8.70775	1.53677	4.76696
N	8.69937	3.42776	3.57762
N	8.71056	4.70891	1.50711
N	8.77992	4.95343	0.71257
N	8.85043	4.26908	3.039
N	8.8983	2.69915	4.63058
N	8.81304	0.3325	5.19889
N	8.79587	1.89125	4.93373
N	6.32088	3.72149	3.36931
N	6.30653	5.0228	1.54936
N	6.37244	5.35652	0.85844
N	6.37579	4.89298	3.04803
N	6.38397	3.13119	4.74015
N	6.32497	0.95294	5.23789
N	6.25219	1.43259	4.77352
N	6.27713	3.10668	3.28203
N	6.27052	4.44644	1.26024
N	6.32816	5.12026	0.87372
N	6.39945	4.42293	3.20851
N	6.43993	2.74216	4.68143
N	6.3614	0.41535	5.39924
N	6.3507	1.72557	4.7491
C	9.54474	4.21878	2.42882
C	9.35225	5.77428	0.51685
H	9.22698	6.85035	0.70993
H	10.41372	5.51759	0.49641
C	9.56462	4.74336	1.7097
C	9.4176	4.17702	4.09793
H	9.35895	4.95556	4.87376
H	10.46233	3.95416	3.86829
C	9.54651	1.76909	4.61737
C	9.29186	0.48486	5.56391
H	10.35813	0.46379	5.32787

N	5.51461	2.02789	4.85993	H	9.1451	0.71371	6.6304
N	5.52105	0.20304	4.75566	C	9.49495	2.34825	3.94829
N	5.5475	2.50656	4.01137	C	9.23033	4.54491	2.84211
N	5.56771	4.39986	2.82308	H	10.30543	4.37922	2.74617
C	8.89975	4.70792	0.35363	H	9.03861	5.46763	3.41016
C	8.79812	4.71589	2.80717	C	9.524	4.54082	0.39227
H	8.7571	5.64084	3.40213	C	9.39424	5.11706	2.00601
H	9.83758	4.43505	2.62202	H	9.30721	6.16758	2.32316
C	8.92667	2.50167	3.89149	H	10.44664	4.84601	1.89347
C	8.65612	0.56485	5.384	C	9.60233	3.22395	3.55556
H	8.48035	0.63179	6.4681	C	9.4346	1.6195	5.41878
H	9.72806	0.53257	5.17859	H	10.48493	1.51459	5.13719
C	8.8978	1.64297	4.29612	H	9.35414	1.88302	6.4841
C	8.67743	4.06157	3.81595	C	9.57112	0.75166	4.75701
H	9.74552	3.89838	3.65803	C	9.32342	3.2085	4.67501
H	8.52132	4.77493	4.63887	H	10.39113	3.08841	4.47915
C	8.92222	4.68735	1.44778	H	9.17016	3.83019	5.56973
C	8.78924	5.77873	0.76015	C	7.4861	4.55708	3.53908
H	9.8222	5.42272	0.74358	H	7.38492	5.16435	4.45233
H	8.76988	6.87429	0.86369	C	7.51466	5.41117	2.22744
C	8.87076	4.29722	2.71815	H	7.50952	6.4965	2.41414
C	8.59452	3.20349	4.90576	C	7.58938	5.97394	1.33754
H	8.44833	3.6931	5.87995	H	7.53395	7.06618	1.21214
H	9.65906	3.03866	4.7255	C	7.65026	5.52606	2.83915
C	8.74352	0.73668	4.85037	H	7.77242	6.36644	3.53934
C	8.44103	1.65187	5.42436	C	7.63819	2.90733	5.41282
H	9.52217	1.59407	5.28205	H	7.73339	3.59385	6.26795
H	8.21075	1.98049	6.44834	C	7.56175	1.39754	5.8351
C	8.77114	3.20559	3.52932	H	7.52749	1.25282	6.92571
C	8.66383	5.17734	2.047	C	7.44254	2.15798	5.11227
H	9.71768	4.89695	1.98363	H	7.43672	2.41974	6.1818
H	8.56581	6.17524	2.50057	C	7.3845	3.40207	4.16878
C	6.98306	5.93678	0.26021	H	7.196	4.34587	4.70318
H	7.02006	6.97931	0.61111	C	7.43689	5.30686	1.19281
C	7.04047	5.80633	1.30314	H	7.28014	6.19984	1.81727
H	7.18796	6.76996	1.81364	C	7.55633	5.63061	0.33121
C	6.99813	3.78386	4.35102	H	7.64342	6.7073	0.54397
H	7.10609	4.63476	5.04079	C	7.68623	4.56954	3.84035
C	6.89368	2.4023	5.08089	H	7.77844	5.57726	4.27271
H	6.78873	2.49324	6.17315	C	7.68865	3.42895	4.91955
C	6.80599	1.16795	5.29212	H	7.71669	3.80872	5.95217
H	6.69267	1.0803	6.3839	C	7.61297	0.08941	5.89369
C	6.81227	2.64556	4.78459	H	7.70845	0.12683	6.96883
H	6.68972	3.38601	5.59056	C	7.5346	1.61966	5.57429

C	6.9165	5.36496	2.47584	H	7.42682	2.24731	6.47246
H	6.81191	6.08692	3.29953	C	5.57817	4.06186	2.2411
C	7.02834	6.04025	1.06708	C	5.75519	5.69553	0.40179
H	7.17281	7.12982	1.11432	H	4.70677	5.39618	0.33007
C	7.02264	5.75552	2.60682	H	5.82826	6.78274	0.55981
H	7.20983	6.81715	2.82395	C	5.60935	4.81346	1.89042
C	6.91211	4.86723	3.89434	C	5.88221	4.41526	4.31648
H	6.85067	5.45212	4.82376	H	6.116	5.1611	5.08814
C	6.70016	1.66562	5.58654	H	4.8005	4.30716	4.2096
H	6.69795	2.14829	6.57563	C	5.58769	1.99135	4.68096
C	6.61392	0.10817	5.65448	C	5.71339	0.32428	5.51411
H	6.39266	0.27425	6.66268	H	5.80207	0.54322	6.58838
C	6.65274	3.25985	4.56908	H	4.66158	0.23299	5.23343
H	6.4274	3.55368	5.60577	C	5.56166	1.98073	3.69468
C	6.7783	4.4718	3.59327	C	5.60765	4.12601	2.50567
H	6.84063	5.44326	4.10639	H	5.52809	5.04226	3.11462
C	4.94878	5.02894	0.52967	H	4.60161	3.76497	2.27003
C	5.25756	5.06421	2.97938	C	5.55753	4.44159	0.06684
H	4.17275	4.95611	2.90209	C	5.85723	5.32248	2.21941
H	5.50792	5.94381	3.5878	H	6.07725	6.35571	2.51924
C	4.95093	2.75562	3.79205	H	4.77816	5.15465	2.20509
C	5.05225	0.64991	5.03353	C	5.64566	3.38082	3.73297
H	5.03457	0.69554	6.13462	C	5.87548	1.75939	5.57505
H	4.03184	0.65407	4.64325	H	6.06181	2.06721	6.61381
C	4.96895	1.50418	3.84976	H	4.80181	1.73465	5.37528
C	5.06424	3.92605	3.47023	C	5.61076	0.54582	4.73199
H	5.03045	4.62433	4.32245	C	5.70955	2.98166	4.44378
H	4.05013	3.68396	3.1443	H	4.68837	2.74981	4.1316
C	4.98012	4.88275	1.21943	H	5.6927	3.60959	5.35015
C	5.28473	6.27828	0.79504	C	7.45555	1.78378	0.40219
H	5.57924	7.33258	0.86018	C	8.56793	1.17847	0.94192
H	4.19654	6.19029	0.77856	C	8.48331	0.14519	1.44097
C	4.9521	4.62751	2.5999	C	7.28838	0.82998	1.40843
C	5.01757	3.36537	4.70117	C	6.11533	0.22532	0.887
H	5.09942	3.88842	5.66501	C	6.21015	1.1088	0.35811
H	3.96941	3.28571	4.40465	N	4.9463	0.93508	0.91394
C	4.82844	0.93263	4.35365	C	3.88595	0.32187	0.42898
C	4.85584	1.48191	4.74883	C	3.88123	0.98596	0.14608
H	4.70842	1.81596	5.79032	C	5.04292	1.70132	0.18902
H	3.88666	1.33871	4.26567	C	2.50903	0.83282	0.38878
C	4.85184	3.23106	3.04356	N	1.73781	0.147	0.20293
C	5.10529	5.40041	1.8965	C	2.50045	1.33931	0.60223
H	4.03594	5.23253	1.75098	C	1.94842	2.00418	0.8257
H	5.27624	6.39056	2.34165	C	0.55012	2.15766	0.63666

O	10.70301	3.90339	2.24926	C	0.21605	1.17006	0.06427
O	10.67886	4.29935	1.53713	C	0.3619	0.07054	0.41386
O	10.65858	1.58382	4.17202	O	0.24931	0.99382	0.95901
O	10.66331	2.18084	3.65582	C	0.17057	3.44796	0.98336
O	10.67791	4.16055	0.39599	C	1.58171	3.13127	1.5198
O	10.6995	2.87737	3.17506	O	2.27624	2.14811	0.97508
O	10.7129	0.7199	4.3485	C	1.69711	1.33307	0.0799
O	4.47963	3.64375	1.94883	O	2.04612	3.80303	2.43024
O	4.47181	4.4053	1.80831	O	0.50956	4.18819	1.97725
O	4.44737	1.93361	4.2753	C	0.28767	4.31385	0.30478
O	4.51356	1.57936	3.24529	C	1.1045	5.59123	0.11109
O	4.45024	3.98482	0.12499	H	7.51227	2.78862	0.00711
O	4.494	3.11557	3.4613	H	9.51058	1.71204	0.98707
O	4.49753	0.39673	4.27709	H	9.36929	0.61593	1.8511
N	8.76432	3.88986	3.53318	H	7.20236	1.84736	1.7729
H	2.19212	0.3644	0.00799	H	5.07368	2.69903	0.61021
H	0.18852	4.41974	2.61569	H	2.09748	2.22965	0.11041
H	0.74599	4.54944	0.58	H	2.43323	1.49104	1.68317
H	0.70892	3.71317	1.11678	H	2.54971	2.76756	1.29486
H	1.03196	6.22764	0.99952	H	1.99059	1.78617	1.03434
H	2.16153	5.36654	0.05929	H	0.72571	6.15622	0.74527

CB7---CPT(H)---CB7:

O	10.01439	0.92895	4.3712	N	8.81468	0.25841	4.96345
O	10.02841	3.86758	1.94777	N	8.84394	1.94282	4.56814
O	10.10891	3.78499	1.83403	N	8.82231	3.84316	3.04378
O	10.20227	0.74267	4.10823	N	8.80716	4.81843	1.03234
O	10.22481	2.95617	3.17588	N	8.74727	4.84516	1.39585
O	10.16865	4.54956	0.27199	N	8.77564	4.02206	3.47908
O	10.076	2.81179	3.63221	N	6.35687	1.95063	4.81554
O	3.97504	1.06478	3.4895	N	6.35718	0.18482	5.48038
O	3.97484	3.95962	1.66568	N	6.3844	2.55808	4.91168
O	4.07248	3.73462	1.41356	N	6.373	4.24354	3.44055
O	4.07892	0.48151	3.4078	N	6.27443	5.3857	1.2925
O	4.11708	3.2955	2.8844	N	6.27938	5.27015	0.93921
O	4.06552	4.83299	0.05991	N	6.31366	4.24208	3.13671
O	3.97613	2.83735	2.81792	N	6.31334	2.66915	4.72576
N	8.04753	0.03041	5.21826	N	6.36491	0.27332	5.1866
N	8.09808	2.21082	4.72104	N	6.3868	1.83339	4.44037
N	8.12605	4.1499	3.24593	N	6.36675	3.73498	2.9377
N	8.25089	5.19498	1.26442	N	6.35255	4.88302	1.02316
N	8.2113	4.97398	1.16726	N	6.29202	5.01267	1.41071

N	8.25181	4.01587	3.19825	N	6.32567	3.86779	3.32724
N	8.22087	1.9224	4.46294	C	9.56347	0.96277	4.80641
N	8.26113	0.27564	4.90698	C	9.42496	1.40323	5.4592
N	8.34808	2.68332	4.5099	H	9.3956	1.65826	6.53051
N	8.39596	4.39869	3.0732	H	10.4616	1.31057	5.12702
N	8.37346	5.45859	0.88849	C	9.57552	3.18032	3.75685
N	8.34938	5.49299	1.35248	C	9.35123	5.30277	2.50938
N	8.16287	4.1317	3.37084	H	9.24959	6.28836	2.98841
N	8.08974	2.3669	4.75247	H	10.40686	5.07147	2.34987
N	5.64206	0.07174	4.77258	C	9.50183	5.15895	0.0574
N	5.66256	2.2887	4.51465	C	9.25144	5.54516	2.37012
N	5.68061	4.33535	3.19418	H	10.32901	5.39173	2.28006
N	5.80935	5.30765	1.1969	H	9.04536	6.52248	2.82714
N	5.77808	5.29549	1.23707	C	9.51927	3.39997	3.58155
N	5.8187	3.85193	2.93455	C	9.38476	1.56198	5.20543
N	5.78419	1.82072	4.24885	H	10.42858	1.51872	4.88559
N	5.81928	0.35942	4.70039	H	9.3313	1.78618	6.28256
N	5.90679	2.73814	4.25968	C	9.59109	0.77487	4.44233
N	5.96185	4.68035	3.16553	C	9.38987	3.23086	4.22101
N	5.94523	5.81849	1.00453	H	9.26153	3.91235	5.07437
N	5.90508	5.48248	1.20138	H	10.45236	3.08077	4.01722
N	5.71207	4.29833	3.30956	C	9.58369	4.04967	1.89638
N	5.6783	2.30603	4.31199	C	9.35037	5.36111	0.18691
C	8.8559	1.04196	4.71426	H	10.40678	5.08391	0.21638
C	8.67	3.50036	4.42344	H	9.24965	6.45729	0.17217
H	8.53709	4.16714	5.28724	C	9.53009	4.15483	2.31822
H	9.73413	3.33859	4.23712	C	9.31732	3.41963	4.67372
C	8.92631	4.33895	2.12266	H	10.39079	3.30445	4.50863
C	8.81879	5.59272	0.00504	H	9.13519	4.09195	5.52313
H	8.75461	6.68586	0.09643	C	7.54387	1.84791	5.64042
H	9.86607	5.28163	0.0085	H	7.43608	2.4797	6.53508
C	8.98687	4.19234	2.03342	C	7.61584	0.31813	5.96591
C	8.77501	3.24869	4.30684	H	7.71542	0.10582	7.0411
H	9.84512	3.12467	4.12826	C	7.65661	3.20032	5.13115
H	8.61153	3.8207	5.23086	H	7.75522	3.48497	6.18998
C	9.03556	0.7859	4.43601	C	7.61406	4.42941	4.16122
C	8.85121	1.55113	5.24961	H	7.60013	5.39715	4.68563
H	9.91783	1.47518	5.02784	C	7.49897	6.04947	0.9276
H	8.70246	1.73809	6.32263	H	7.53211	7.0529	1.37831
C	9.11649	3.29606	3.52662	C	7.44256	6.075	0.63809
C	8.99302	5.37377	2.19324	H	7.31796	7.08671	1.05331
H	8.96475	6.36397	2.67064	C	7.48882	4.54721	3.92897
H	10.02906	5.06512	2.03455	H	7.3606	5.51445	4.43772
C	9.08422	5.08577	0.2468	C	7.57467	3.33461	4.91457

C	8.81768	5.23505	2.69207
H	9.87496	4.97288	2.61214
H	8.70207	6.14933	3.29052
C	8.91377	3.06586	3.87747
C	8.58333	1.26974	5.5498
H	9.66174	1.21878	5.38619
H	8.37254	1.48011	6.60776
C	6.74187	0.49841	5.61047
H	6.54191	0.23876	6.65994
C	6.80932	2.03945	5.35112
H	6.74665	2.64347	6.26759
C	6.96138	5.00722	3.24112
H	6.99261	5.7036	4.08962
C	7.03649	5.71815	1.84228
H	7.07841	6.81347	1.90898
C	7.06052	5.53775	1.84988
H	7.20022	6.61264	2.02574
C	6.98384	4.69179	3.16532
H	6.84144	5.29731	4.07058
C	6.94663	1.67708	5.09479
H	6.83795	2.30553	5.99092
C	6.9753	0.14714	5.40635
H	6.88779	0.08447	6.47794
C	7.11018	3.37386	4.76261
H	7.00267	3.59071	5.83478
C	7.21899	4.65704	3.87275
H	7.34091	5.58369	4.45092
C	7.23694	6.29706	0.57251
H	7.40024	7.32025	0.93758
C	7.14726	6.19841	0.99132
H	7.10915	7.17738	1.48874
C	6.91603	4.30347	4.09653
H	6.96022	5.21037	4.71597
C	6.80115	2.98534	4.92786
H	6.60209	3.15661	5.99568
C	4.9842	1.12903	4.16344
C	5.09214	3.58705	4.27851
H	4.04264	3.43782	4.01802
H	5.17129	4.18154	5.19748
C	5.03892	4.46983	1.98222
C	5.2956	5.89128	0.01962
H	5.54171	6.95878	0.03203
H	4.21242	5.75943	0.00814
C	5.11579	4.22747	1.81058
H	7.68861	3.6349	5.96691
C	7.63454	0.2291	5.64536
H	7.73069	0.06657	6.72945
C	7.59029	1.74025	5.24591
H	7.51392	2.41823	6.10929
C	7.56463	4.54651	3.0202
H	7.49464	5.22475	3.88359
C	7.58829	5.29115	1.64504
H	7.61185	6.38705	1.74007
C	7.55064	5.39176	1.99897
H	7.62158	6.48837	2.05397
C	7.50972	4.70079	3.40349
H	7.41266	5.4132	4.23657
C	5.60582	0.77802	4.82038
C	5.84225	1.50735	5.73642
H	4.7695	1.47384	5.53464
H	6.02251	1.75367	6.7922
C	5.60523	3.20666	3.96098
C	5.76129	5.27286	2.63215
H	5.87586	6.24383	3.13664
H	4.70307	5.01637	2.5468
C	5.54588	4.9427	0.19574
C	5.6837	5.18908	2.24851
H	5.69805	6.18775	2.71339
H	4.65256	4.85529	2.1102
C	5.57034	3.19705	3.67639
C	5.8225	1.56946	5.51409
H	6.02388	1.7769	6.57318
H	4.7463	1.5129	5.33861
C	5.62444	0.67288	4.49404
C	5.77115	3.08826	4.08112
H	5.8212	3.77436	4.94111
H	4.72571	2.88432	3.83828
C	5.62641	3.98628	1.79319
C	5.79154	5.5007	0.15214
H	5.96955	6.583	0.09822
H	4.72156	5.30029	0.13832
C	5.5659	4.1287	2.19474
C	5.73979	3.2062	4.46754
H	4.70066	2.98953	4.20985
H	5.77929	3.88313	5.33594
C	6.71506	1.3231	1.05121
C	7.76214	0.56226	1.52618
C	7.6682	0.84814	1.52812

C	5.16226	3.08123	3.9646
H	5.12075	3.68079	4.88636
H	4.15044	2.87222	3.61157
C	5.11655	0.62567	4.03034
C	5.24244	1.65589	4.93764
H	5.2443	1.85094	6.02055
H	4.21556	1.62822	4.56902
C	5.2091	3.5351	3.36579
C	5.47605	5.77914	2.36805
H	5.75088	6.71786	2.8623
H	4.3899	5.68338	2.32057
C	5.18124	5.31062	0.03054
C	5.25009	5.37996	2.4837
H	5.37781	6.32219	3.03277
H	4.19171	5.20026	2.28453
C	5.00946	3.10791	3.39791
C	4.98726	1.20132	4.92504
H	4.01331	1.12453	4.43746
H	4.85288	1.40761	5.99887
O	10.69558	0.91734	4.3773
O	10.68697	2.90127	3.36127
O	10.6568	4.79457	0.01577
O	10.65292	3.1674	3.22128
O	10.72205	0.68784	4.01184
O	10.72306	3.67674	1.71066
O	10.67399	3.79181	2.15395
O	4.4822	0.64048	4.38276
O	4.45243	2.95084	3.68133
O	4.45032	4.42239	0.22333
O	4.45972	2.8481	3.3372
O	4.49656	0.52908	4.06489
O	4.52266	3.54458	1.53162
O	4.44826	3.70881	1.96267
N	8.80383	2.11131	4.9987
N	8.80484	0.11293	5.26062
N	8.83147	2.47736	4.70212
N	8.82988	4.29817	3.40144
N	8.73126	5.34148	1.204
N	8.71988	5.53608	1.02779
N	8.75535	4.51268	3.24723
N	8.76094	2.63359	4.46476

C	6.52086	1.48219	1.0917
C	5.43699	0.7138	0.62478
C	5.53076	0.71047	0.56842
N	4.2754	1.31996	0.19083
C	3.28005	0.61482	0.34802
C	3.36598	0.78944	0.4804
C	4.45872	1.45905	0.00481
C	2.00192	1.0726	0.85156
N	1.34852	0.05262	1.31899
C	2.13202	1.28666	1.1755
C	1.41819	2.30886	0.90311
C	0.10614	2.38072	1.4441
C	0.54301	1.2694	1.91769
C	0.08057	0.05139	1.90982
O	0.40448	1.08656	2.35541
C	0.63443	3.6989	1.471
C	1.52295	3.771	2.72743
O	2.10587	2.66406	3.17974
C	1.92271	1.39779	2.48899
O	1.66293	4.83217	3.30837
O	0.30078	4.76218	1.55159
C	1.5066	3.84123	0.18999
C	2.06304	5.25061	0.01178
H	6.78666	2.40608	1.02355
H	8.67278	1.03484	1.87853
H	8.51768	1.43518	1.86012
H	6.4612	2.56269	1.07569
H	4.50562	2.54196	0.03927
H	1.57194	2.03093	0.60228
H	2.36721	1.7175	2.15271
H	1.93066	3.19799	0.56811
H	2.1256	0.62533	3.22804
H	2.70029	1.32741	1.7214
H	0.11249	5.39196	2.16921
H	2.32826	3.12109	0.20442
H	0.86268	3.58413	0.65809
H	1.25452	5.98208	0.06208
H	2.70096	5.52562	0.85611
H	2.65779	5.28269	0.90373
H	4.17007	2.33345	0.29681

Au-CB7---CPT---CB7-Au:

Au	1.44188	0.83247	1.17729	N	3.73294	7.69995	27.34021
Au	4.32564	0.83247	1.17729	C	4.62026	5.4745	27.59525
Au	7.2094	0.83247	1.17729	H	3.97145	9.78423	27.74
Au	10.09315	0.83247	1.17729	H	2.31403	9.14813	27.98245
Au	12.97691	0.83247	1.17729	C	3.39042	8.90869	28.09806
Au	15.86067	0.83247	1.17729	C	4.24822	6.69622	28.12401
Au	0	3.32988	1.17729	H	5.03518	4.68706	28.22906
Au	2.88376	3.32988	1.17729	O	1.53427	11.49827	28.865
Au	5.76752	3.32988	1.17729	O	0.34374	7.96252	28.85843
Au	8.65128	3.32988	1.17729	O	1.57586	3.99911	28.8751
Au	11.53503	3.32988	1.17729	O	7.3096	4.90161	28.90462
Au	14.41879	3.32988	1.17729	O	4.57338	2.5208	28.91812
Au	-1.44188	5.82729	1.17729	O	4.70687	11.94982	28.92539
Au	1.44188	5.82729	1.17729	H	-0.07789	5.65061	28.96711
Au	4.32564	5.82729	1.17729	H	-0.42959	10.25818	28.97832
Au	7.2094	5.82729	1.17729	O	7.06193	8.90267	28.98117
Au	10.09315	5.82729	1.17729	H	8.38051	6.92181	29.09863
Au	12.97691	5.82729	1.17729	H	6.92047	11.28031	29.11103
Au	-2.88376	8.32469	1.17729	H	2.39418	1.70314	29.19549
Au	0	8.32469	1.17729	H	2.87202	13.45739	29.24082
Au	2.88376	8.32469	1.17729	H	6.9162	2.57184	29.23888
Au	5.76752	8.32469	1.17729	C	4.30188	7.19092	29.4981
Au	8.65128	8.32469	1.17729	C	3.76101	8.51833	29.49744
Au	11.53503	8.32469	1.17729	H	-1.66201	5.1296	29.71718
Au	-4.32564	10.8221	1.17729	C	-0.31904	7.92012	29.88584
Au	-1.44188	10.8221	1.17729	C	-0.62194	5.48977	29.92487
Au	1.44188	10.8221	1.17729	C	1.06738	3.72104	29.95141
Au	4.32564	10.8221	1.17729	C	1.16645	11.79729	29.99081
Au	7.2094	10.8221	1.17729	C	-0.80228	10.34104	30.02288
Au	10.09315	10.8221	1.17729	C	7.65482	9.09679	30.03148
Au	-5.76752	13.31951	1.17729	C	7.73087	4.78258	30.0441
Au	-2.88376	13.31951	1.17729	C	4.94039	12.33737	30.05951
Au	0	13.31951	1.17729	H	9.96606	6.94693	30.01521
Au	2.88376	13.31951	1.17729	H	-1.81932	10.80043	30.0325
Au	5.76752	13.31951	1.17729	C	4.63248	2.17583	30.08981
Au	8.65128	13.31951	1.17729	C	8.85075	6.94883	30.10734
Au	0	1.66494	3.53187	C	7.24762	11.5242	30.14645
Au	2.88376	1.66494	3.53187	H	8.07795	12.27223	30.12498
Au	5.76752	1.66494	3.53187	C	2.25796	1.57729	30.29153
Au	8.65128	1.66494	3.53187	C	7.07365	2.42267	30.33194
Au	11.53503	1.66494	3.53187	C	2.83071	13.58202	30.34499
Au	14.41879	1.66494	3.53187	H	7.76486	1.56514	30.51251
Au	-1.44188	4.16235	3.53187	H	1.84771	0.56665	30.51811

Au	1.44188	4.16235	3.53187	N	-0.89195	9.0053	30.54414
Au	4.32564	4.16235	3.53187	N	4.76659	6.52017	30.53808
Au	7.2094	4.16235	3.53187	N	-0.68803	6.77091	30.58902
Au	10.09315	4.16235	3.53187	N	0.10957	4.47393	30.64326
Au	12.97691	4.16235	3.53187	H	2.68173	14.65154	30.61326
Au	-2.88376	6.65975	3.53187	N	0.12818	11.20836	30.7104
Au	0	6.65975	3.53187	N	1.29394	2.56754	30.70291
Au	2.88376	6.65975	3.53187	C	3.67743	9.18566	30.70027
Au	5.76752	6.65975	3.53187	N	7.72852	10.30335	30.72931
Au	8.65128	6.65975	3.53187	N	8.43355	8.17637	30.7332
Au	11.53503	6.65975	3.53187	N	1.68324	12.82265	30.78217
Au	-4.32564	9.15716	3.53187	H	3.25697	10.19591	30.78809
Au	-1.44188	9.15716	3.53187	N	8.41464	5.7511	30.77987
Au	1.44188	9.15716	3.53187	N	6.09541	12.10038	30.80025
Au	4.32564	9.15716	3.53187	N	4.11159	13.1413	30.84264
Au	7.2094	9.15716	3.53187	N	7.68497	3.63055	30.82727
Au	10.09315	9.15716	3.53187	N	3.57477	1.70422	30.86226
Au	-5.76752	11.65457	3.53187	N	5.77504	2.11675	30.8828
Au	-2.88376	11.65457	3.53187	H	-2.63611	6.61627	31.39449
Au	0	11.65457	3.53187	H	-1.50284	3.4123	31.51904
Au	2.88376	11.65457	3.53187	C	-1.64154	7.05961	31.64481
Au	5.76752	11.65457	3.53187	H	-2.68536	9.06197	31.63933
Au	8.65128	11.65457	3.53187	C	-1.65803	8.62761	31.70084
Au	-7.2094	14.15198	3.53187	H	10.22839	8.69689	31.70984
Au	-4.32564	14.15198	3.53187	C	4.71015	7.19006	31.726
Au	-1.44188	14.15198	3.53187	C	-0.45959	3.73309	31.75718
Au	1.44188	14.15198	3.53187	H	10.17466	5.16841	31.8005
Au	4.32564	14.15198	3.53187	H	9.39823	11.00683	31.80075
Au	7.2094	14.15198	3.53187	H	-1.16157	12.44798	31.83781
Au	0	0	5.88645	C	4.1658	8.5231	31.86173
Au	2.88376	0	5.88645	C	9.12291	8.76444	31.85691
Au	5.76752	0	5.88645	C	8.58928	10.24055	31.88176
Au	8.65128	0	5.88645	C	-0.17737	11.92951	31.93273
Au	11.53503	0	5.88645	C	0.53629	2.53398	31.92325
Au	14.41879	0	5.88645	H	6.94299	13.67241	31.9333
Au	-1.44188	2.49741	5.88645	C	9.0672	5.19252	31.94525
Au	1.44188	2.49741	5.88645	C	6.15136	12.88831	32.01659
Au	4.32564	2.49741	5.88645	H	0.02385	1.54636	32.02923
Au	7.2094	2.49741	5.88645	C	1.03606	12.92113	32.06597
Au	10.09315	2.49741	5.88645	C	8.42465	3.75952	32.05453
Au	12.97691	2.49741	5.88645	C	4.69675	13.48361	32.11474
Au	-2.88376	4.99482	5.88645	H	9.18135	2.94161	32.1359
Au	0	4.99482	5.88645	C	3.9823	1.24332	32.16802
Au	2.88376	4.99482	5.88645	C	5.53595	1.51593	32.17519

Au	5.76752	4.99482	5.88645	H	0.72085	13.97562	32.25662
Au	8.65128	4.99482	5.88645	H	4.68304	14.58979	32.258
Au	11.53503	4.99482	5.88645	H	3.72448	0.16484	32.2919
Au	-4.32564	7.49222	5.88645	H	6.14374	0.58508	32.2856
Au	-1.44188	7.49222	5.88645	H	5.55534	5.49448	32.76995
Au	1.44188	7.49222	5.88645	C	5.19033	6.52524	32.89099
Au	4.32564	7.49222	5.88645	N	-1.25025	6.68132	32.97747
Au	7.2094	7.49222	5.88645	N	-1.07016	8.92277	32.99202
Au	10.09315	7.49222	5.88645	N	-0.42157	4.38007	33.04381
Au	-5.76752	9.98963	5.88645	N	1.23604	2.86835	33.15042
Au	-2.88376	9.98963	5.88645	C	4.13246	9.12741	33.14571
Au	0	9.98963	5.88645	N	-0.12893	11.17582	33.15962
Au	2.88376	9.98963	5.88645	N	8.76738	8.25166	33.15849
Au	5.76752	9.98963	5.88645	N	7.93935	10.34173	33.16878
Au	8.65128	9.98963	5.88645	N	1.76031	12.39672	33.20752
Au	-7.2094	12.48704	5.88645	N	8.7521	5.80255	33.21409
Au	-4.32564	12.48704	5.88645	H	3.67868	10.12116	33.25904
Au	-1.44188	12.48704	5.88645	N	6.30334	12.16198	33.25371
Au	1.44188	12.48704	5.88645	N	4.15638	12.82174	33.2848
Au	4.32564	12.48704	5.88645	H	-2.40736	4.96894	33.29274
Au	7.2094	12.48704	5.88645	N	7.65781	3.84251	33.28165
Au	1.44188	0.83247	8.24103	N	3.50759	1.99845	33.30543
Au	4.32564	0.83247	8.24103	N	5.72108	2.36461	33.32902
Au	7.2094	0.83247	8.24103	H	-2.13477	10.67938	33.4409
Au	10.09315	0.83247	8.24103	C	-1.38271	5.34728	33.51878
Au	12.97691	0.83247	8.24103	H	10.41391	7.01986	33.56091
Au	15.86067	0.83247	8.24103	C	-1.12745	10.23203	33.60967
Au	0	3.32988	8.24103	H	8.34832	12.34711	33.64402
Au	2.88376	3.32988	8.24103	C	9.31041	7.03556	33.72266
Au	5.76752	3.32988	8.24103	H	1.77132	0.93509	33.79898
Au	8.65128	3.32988	8.24103	C	7.53299	11.59521	33.76476
Au	11.53503	3.32988	8.24103	C	-0.91629	7.77472	33.77351
Au	14.41879	3.32988	8.24103	C	2.16011	1.98038	33.82358
Au	-1.44188	5.82729	8.24103	H	2.66124	14.1801	33.87705
Au	1.44188	5.82729	8.24103	C	2.85655	13.08249	33.8627
Au	4.32564	5.82729	8.24103	H	7.66212	1.83335	33.91806
Au	7.2094	5.82729	8.24103	C	0.59488	3.88523	33.85679
Au	10.09315	5.82729	8.24103	C	6.99125	2.72451	33.91288
Au	12.97691	5.82729	8.24103	C	1.02396	11.42688	33.89621
Au	-2.88376	8.32469	8.24103	C	8.09452	9.18876	33.93458
Au	0	8.32469	8.24103	C	5.12657	12.11465	33.98811
Au	2.88376	8.32469	8.24103	C	7.91956	5.00985	33.98834
Au	5.76752	8.32469	8.24103	C	4.52696	2.65587	33.98082
Au	8.65128	8.32469	8.24103	C	5.16417	7.15238	34.12603

Au	11.53503	8.32469	8.24103	C	4.64196	8.46748	34.25223
Au	-4.32564	10.8221	8.24103	H	-1.25992	5.42888	34.62232
Au	-1.44188	10.8221	8.24103	H	-0.97989	10.0831	34.70324
Au	1.44188	10.8221	8.24103	H	9.10446	7.06024	34.81702
Au	4.32564	10.8221	8.24103	H	7.37975	11.4008	34.85086
Au	7.2094	10.8221	8.24103	H	2.21483	2.31719	34.88405
Au	10.09315	10.8221	8.24103	H	2.89084	12.71352	34.91267
Au	-5.76752	13.31951	8.24103	H	6.79243	3.02828	34.96738
Au	-2.88376	13.31951	8.24103	O	-0.57509	7.741	34.94397
Au	0	13.31951	8.24103	H	5.54107	6.63075	35.02005
Au	2.88376	13.31951	8.24103	O	0.8631	4.2569	34.99028
Au	5.76752	13.31951	8.24103	O	1.32977	10.90869	34.95836
Au	8.65128	13.31951	8.24103	O	4.40472	3.34801	34.98348
Au	-0.00887	1.65962	10.69901	O	4.9741	11.55754	35.06683
Au	2.8814	1.65308	10.68594	O	7.73146	9.04061	35.09384
Au	5.77274	1.65523	10.68851	O	7.50635	5.28488	35.10848
Au	8.65768	1.6614	10.69684	H	4.63176	8.97036	35.2328
Au	11.53708	1.66495	10.71352	Au	-0.00152	0.00253	37.48604
Au	14.41524	1.66698	10.70757	Au	2.88117	-0.00679	37.47652
Au	-1.45121	4.15557	10.69293	Au	5.7698	-0.01076	37.49353
Au	1.44141	4.15546	10.68133	Au	8.65129	-0.00376	37.4863
Au	4.32845	4.15282	10.68364	Au	11.53394	0.00705	37.46636
Au	7.21479	4.15755	10.69007	Au	14.41915	0.00796	37.50662
Au	10.10065	4.1577	10.69795	Au	-1.44981	2.49702	37.5006
Au	12.97378	4.15975	10.71061	Au	1.43095	2.48371	37.55915
Au	-2.89208	6.65288	10.69701	Au	4.32158	2.47012	37.56724
Au	-0.00179	6.66029	10.67952	Au	7.21951	2.47831	37.56332
Au	2.8877	6.65643	10.68154	Au	10.09968	2.48859	37.49745
Au	5.77074	6.65741	10.68918	Au	12.97542	2.49633	37.51031
Au	8.65846	6.65739	10.68817	Au	-2.8937	4.98973	37.48686
Au	11.5398	6.65635	10.69911	Au	-0.02017	4.99885	37.56679
Au	-4.326	9.16027	10.71285	Au	2.87417	4.98818	37.54655
Au	-1.45333	9.16062	10.68215	Au	5.76009	4.97618	37.59728
Au	1.4384	9.16101	10.68176	Au	8.68072	4.98056	37.56943
Au	4.33043	9.15511	10.68318	Au	11.54272	4.99305	37.50024
Au	7.22032	9.15917	10.68201	Au	-4.33134	7.49457	37.5088
Au	10.10316	9.15719	10.69154	Au	-1.46702	7.49088	37.54842
Au	-5.76679	11.65774	10.71555	Au	1.44011	7.493	37.54491
Au	-2.88707	11.65659	10.709	Au	4.31979	7.4796	37.58916
Au	-0.00564	11.66429	10.68715	Au	7.21508	7.47758	37.59127
Au	2.88366	11.65695	10.68219	Au	10.10428	7.49448	37.53307
Au	5.77329	11.65985	10.6848	Au	-5.75956	9.99515	37.50538
Au	8.65908	11.66095	10.69036	Au	-2.88751	9.99563	37.48434
Au	-7.20954	14.14966	10.71674	Au	-0.00729	9.98805	37.55916

Au	-4.3273	14.14835	10.71421
Au	-1.44303	14.14958	10.71264
Au	1.4444	14.16185	10.69961
Au	4.32594	14.16254	10.69387
Au	7.20943	14.1598	10.70191
Au	-0.00218	0.0006	13.29361
Au	2.87952	-0.0135	13.29636
Au	5.77018	-0.01453	13.27161
Au	8.64986	0.0001	13.2734
Au	11.53624	0.01038	13.30417
Au	14.41934	0.00831	13.26947
Au	-1.44913	2.49647	13.27852
Au	1.43193	2.47746	13.19354
Au	4.3216	2.46034	13.17929
Au	7.21713	2.47972	13.22011
Au	10.10009	2.48944	13.27186
Au	12.97594	2.49726	13.26612
Au	-2.8967	4.98852	13.28411
Au	-0.01906	4.99657	13.16631
Au	2.89073	4.9879	13.19796
Au	5.77045	4.97975	13.19828
Au	8.68222	4.9767	13.20514
Au	11.54244	4.9935	13.28153
Au	-4.3333	7.49439	13.27165
Au	-1.47162	7.49105	13.17898
Au	1.46081	7.48858	13.19014
Au	4.33832	7.48166	13.24629
Au	7.21901	7.47437	13.19252
Au	10.10665	7.49533	13.23019
Au	-5.75875	9.99523	13.27205
Au	-2.88631	9.99577	13.29205
Au	-0.00378	9.98927	13.16885
Au	2.90801	9.98411	13.18959
Au	5.78567	9.97707	13.18563
Au	8.67732	10.00693	13.19601
Au	-7.20484	12.4923	13.30409
Au	-4.32473	12.48595	13.26803
Au	-1.44351	12.48215	13.289
Au	1.43789	12.51077	13.19567
Au	4.32244	12.5113	13.1782
Au	7.21929	12.50177	13.21343
O	1.55028	10.9503	15.58732
O	-0.27434	7.74081	15.61101
O	5.20133	11.50334	15.61754

Au	2.89025	9.99173	37.56469
Au	5.78084	9.98882	37.60946
Au	8.67567	10.0079	37.56242
Au	-7.20475	12.49485	37.48408
Au	-4.32521	12.48802	37.50642
Au	-1.44459	12.48192	37.48379
Au	1.4362	12.50096	37.54538
Au	4.32063	12.51118	37.57877
Au	7.2175	12.50078	37.53381
Au	-0.00604	1.66171	40.07395
Au	2.88075	1.65609	40.08114
Au	5.76874	1.65678	40.08233
Au	8.65869	1.6579	40.0779
Au	11.5375	1.66328	40.06127
Au	14.41692	1.66699	40.06998
Au	-1.44762	4.15866	40.07434
Au	1.43706	4.15752	40.08011
Au	4.32076	4.15369	40.0888
Au	7.2158	4.15557	40.0895
Au	10.10186	4.15553	40.07588
Au	12.97735	4.16095	40.06671
Au	-2.88713	6.6562	40.06956
Au	-0.00559	6.66095	40.07614
Au	2.87571	6.65442	40.09307
Au	5.77014	6.6585	40.09965
Au	8.66057	6.65716	40.08695
Au	11.54097	6.65729	40.07523
Au	-4.32388	9.16035	40.06598
Au	-1.44892	9.15887	40.07564
Au	1.43548	9.15893	40.08154
Au	4.3228	9.16031	40.10073
Au	7.2205	9.16069	40.09573
Au	10.10225	9.15786	40.07811
Au	-5.7659	11.65765	40.06288
Au	-2.88481	11.65584	40.06595
Au	-0.00469	11.65963	40.07659
Au	2.8802	11.65848	40.08374
Au	5.77146	11.6654	40.08951
Au	8.65664	11.66103	40.07906
Au	-7.20816	14.15105	40.06037
Au	-4.32602	14.14957	40.06004
Au	-1.44258	14.1494	40.05934
Au	1.44248	14.1571	40.06786
Au	4.32458	14.16161	40.07436

O	1.04208	4.36322	15.63363	Au	7.21039	14.15892	40.07014
O	4.52628	3.3288	15.67825	Au	1.44188	0.83247	42.5344
O	7.85173	9.05141	15.66094	Au	4.32564	0.83247	42.5344
O	7.597	5.37488	15.66667	Au	7.2094	0.83247	42.5344
H	-1.1327	5.49105	15.73827	Au	10.09315	0.83247	42.5344
H	7.59804	11.44051	15.74995	Au	12.97691	0.83247	42.5344
H	3.13087	12.76705	15.77692	Au	15.86067	0.83247	42.5344
H	2.33297	2.34163	15.78564	Au	0	3.32988	42.5344
H	-0.72945	10.12823	15.80128	Au	2.88376	3.32988	42.5344
H	9.25592	7.08638	15.86151	Au	5.76752	3.32988	42.5344
H	6.92339	3.06142	15.92183	Au	8.65128	3.32988	42.5344
C	0.60236	3.91188	16.68001	Au	11.53503	3.32988	42.5344
C	-0.81346	7.77189	16.70761	Au	14.41879	3.32988	42.5344
C	1.28165	11.35878	16.70839	Au	-1.44188	5.82729	42.5344
C	5.37308	12.06315	16.69075	Au	1.44188	5.82729	42.5344
C	8.40369	9.28505	16.72608	Au	4.32564	5.82729	42.5344
C	4.57561	2.79664	16.78012	Au	7.2094	5.82729	42.5344
C	8.00019	5.15377	16.80305	Au	10.09315	5.82729	42.5344
C	-1.39596	5.36948	16.8126	Au	12.97691	5.82729	42.5344
C	7.81093	11.68162	16.8161	Au	-2.88376	8.32469	42.5344
C	3.10136	13.03336	16.85717	Au	0	8.32469	42.5344
C	2.21933	2.05392	16.85578	Au	2.88376	8.32469	42.5344
C	-0.92783	10.24332	16.89107	Au	5.76752	8.32469	42.5344
H	8.54583	12.52021	16.85035	Au	8.65128	8.32469	42.5344
H	-2.45518	5.02826	16.87768	Au	11.53503	8.32469	42.5344
C	9.52288	7.0925	16.94338	Au	-4.32564	10.8221	42.5344
H	1.93309	0.97671	16.90458	Au	-1.44188	10.8221	42.5344
H	2.87314	14.12104	16.94811	Au	1.44188	10.8221	42.5344
C	7.03968	2.87893	17.01577	Au	4.32564	10.8221	42.5344
H	-1.87882	10.81178	17.01522	Au	7.2094	10.8221	42.5344
H	10.62509	6.94926	17.04215	Au	10.09315	10.8221	42.5344
H	7.67045	1.97167	17.16858	Au	-5.76752	13.31951	42.5344
N	-0.53771	4.33943	17.35061	Au	-2.88376	13.31951	42.5344
N	4.41972	12.78649	17.39651	Au	0	13.31951	42.5344
N	8.40118	10.50059	17.40029	Au	2.88376	13.31951	42.5344
N	1.14937	2.85836	17.40508	Au	5.76752	13.31951	42.5344
N	6.56222	12.10831	17.41198	Au	8.65128	13.31951	42.5344
N	-1.2719	6.67366	17.431	Au	0	0	44.88897
N	-1.10285	8.9166	17.44072	Au	2.88376	0	44.88897
N	0.15883	11.01557	17.45202	Au	5.76752	0	44.88897
N	2.02706	12.2672	17.45434	Au	8.65128	0	44.88897
N	9.16966	8.39319	17.46604	Au	11.53503	0	44.88897
N	3.50907	2.24408	17.47955	Au	14.41879	0	44.88897
N	5.72043	2.62125	17.54697	Au	-1.44188	2.49741	44.88897

N	8.83414	5.97601	17.55141	Au	1.44188	2.49741	44.88897
N	7.7269	4.0246	17.56264	Au	4.32564	2.49741	44.88897
H	-0.04097	1.47554	18.46371	Au	7.2094	2.49741	44.88897
H	7.138	13.8186	18.50265	Au	10.09315	2.49741	44.88897
H	-3.03803	6.76188	18.57921	Au	12.97691	2.49741	44.88897
H	9.99283	11.22968	18.57094	Au	-2.88376	4.99482	44.88897
C	0.35925	2.51377	18.56788	Au	0	4.99482	44.88897
C	-0.7679	3.61299	18.57678	Au	2.88376	4.99482	44.88897
H	-1.79901	3.18597	18.57968	Au	5.76752	4.99482	44.88897
C	6.45972	12.93477	18.5964	Au	8.65128	4.99482	44.88897
H	3.79461	0.48607	18.61553	Au	11.53503	4.99482	44.88897
C	9.17683	10.4693	18.61826	Au	-4.32564	7.49222	44.88897
C	-1.96366	7.06282	18.64169	Au	-1.44188	7.49222	44.88897
C	4.93449	13.32685	18.63278	Au	1.44188	7.49222	44.88897
H	10.8263	8.9218	18.6537	Au	4.32564	7.49222	44.88897
H	1.12988	13.72067	18.68724	Au	7.2094	7.49222	44.88897
C	9.71116	8.98725	18.6681	Au	10.09315	7.49222	44.88897
H	4.76443	14.42976	18.6669	Au	-5.76752	9.98963	44.88897
C	1.3729	12.63027	18.69334	Au	-2.88376	9.98963	44.88897
C	-1.75878	8.62262	18.69124	Au	0	9.98963	44.88897
C	3.93297	1.59018	18.70285	Au	2.88376	9.98963	44.88897
C	0.09694	11.71062	18.71607	Au	5.76752	9.98963	44.88897
H	-2.71769	9.19144	18.76151	Au	8.65128	9.98963	44.88897
H	10.31986	5.13131	18.79045	Au	-7.2094	12.48704	44.88897
H	-0.85886	12.28426	18.78639	Au	-4.32564	12.48704	44.88897
C	9.22739	5.3632	18.80726	Au	-1.44188	12.48704	44.88897
C	5.43761	2.02131	18.82591	Au	1.44188	12.48704	44.88897
C	8.32112	4.08279	18.87544	Au	4.32564	12.48704	44.88897
H	6.12939	1.16439	19.01019	Au	7.2094	12.48704	44.88897
H	8.89464	3.14582	19.07672	Au	0	1.66494	47.24355
N	-0.52215	4.33328	19.80934	Au	2.88376	1.66494	47.24355
N	4.46081	12.70748	19.85109	Au	5.76752	1.66494	47.24355
N	8.44236	10.60349	19.85404	Au	8.65128	1.66494	47.24355
N	0.987	2.67535	19.85467	Au	11.53503	1.66494	47.24355
N	6.66543	12.28116	19.86254	Au	14.41879	1.66494	47.24355
N	-1.38875	6.61404	19.88274	Au	-1.44188	4.16235	47.24355
N	-0.96901	8.81787	19.88665	Au	1.44188	4.16235	47.24355
N	0.28721	10.90225	19.89718	Au	4.32564	4.16235	47.24355
N	2.06211	12.27671	19.90723	Au	7.2094	4.16235	47.24355
N	3.35763	2.07351	19.92572	Au	10.09315	4.16235	47.24355
N	9.19773	8.49262	19.9213	Au	12.97691	4.16235	47.24355
N	5.44097	2.92092	19.9681	Au	-2.88376	6.65975	47.24355
N	7.41323	4.36053	19.97197	Au	0	6.65975	47.24355
N	8.87885	6.07165	20.00336	Au	2.88376	6.65975	47.24355

H	1.7574	0.75882	20.19579	Au	5.76752	6.65975	47.24355
H	8.6917	12.65094	20.23645	Au	8.65128	6.65975	47.24355
H	-2.49133	4.88367	20.28638	Au	11.53503	6.65975	47.24355
H	2.98106	14.08922	20.41146	Au	-4.32564	9.15716	47.24355
H	10.65759	7.07469	20.43273	Au	-1.44188	9.15716	47.24355
C	7.9354	11.84464	20.38943	Au	1.44188	9.15716	47.24355
C	-1.45083	5.27137	20.39912	Au	4.32564	9.15716	47.24355
C	2.01836	1.82326	20.39621	Au	7.2094	9.15716	47.24355
C	3.18262	12.99198	20.46317	Au	10.09315	9.15716	47.24355
H	-1.70494	10.67948	20.51759	Au	-5.76752	11.65457	47.24355
C	-0.74717	10.10304	20.50617	Au	-2.88376	11.65457	47.24355
C	9.55657	7.23006	20.5238	Au	0	11.65457	47.24355
C	0.45207	3.72262	20.60478	Au	2.88376	11.65457	47.24355
C	-0.83814	7.64906	20.64063	Au	5.76752	11.65457	47.24355
C	6.66909	3.30998	20.63589	Au	8.65128	11.65457	47.24355
C	5.50087	12.18698	20.62789	Au	-7.2094	14.15198	47.24355
C	1.39051	11.3077	20.65573	Au	-4.32564	14.15198	47.24355
C	4.23963	2.85462	20.67653	Au	-1.44188	14.15198	47.24355
C	8.53042	9.4678	20.66365	Au	1.44188	14.15198	47.24355
C	7.80818	5.49463	20.69964	Au	4.32564	14.15198	47.24355
H	7.32589	2.40813	20.72438	Au	7.2094	14.15198	47.24355
H	-1.17403	5.34191	21.47485	Au	1.44188	0.83247	49.59813
H	7.77025	11.66397	21.47535	Au	4.32564	0.83247	49.59813
H	2.03133	2.02238	21.49011	Au	7.2094	0.83247	49.59813
H	3.26894	12.66099	21.52272	Au	10.09315	0.83247	49.59813
H	-0.40772	9.89331	21.54559	Au	12.97691	0.83247	49.59813
H	9.2584	7.31362	21.59259	Au	15.86067	0.83247	49.59813
H	6.41848	3.66295	21.66421	Au	0	3.32988	49.59813
O	7.34832	5.89743	21.74848	Au	2.88376	3.32988	49.59813
O	3.98615	3.34563	21.7642	Au	5.76752	3.32988	49.59813
O	-0.3706	7.55423	21.76036	Au	8.65128	3.32988	49.59813
O	1.68648	10.91717	21.76826	Au	11.53503	3.32988	49.59813
O	0.74764	4.01975	21.75085	Au	14.41879	3.32988	49.59813
O	5.41703	11.76926	21.7668	Au	-1.44188	5.82729	49.59813
O	8.13716	9.36454	21.81058	Au	1.44188	5.82729	49.59813
O	5.01284	5.36017	23.41494	Au	4.32564	5.82729	49.59813
H	3.99999	7.15088	23.42957	Au	7.2094	5.82729	49.59813
H	2.96804	5.69178	23.54946	Au	10.09315	5.82729	49.59813
H	4.225	1.86677	23.72414	Au	12.97691	5.82729	49.59813
O	6.42078	3.66843	23.75366	Au	-2.88376	8.32469	49.59813
C	3.90829	6.16132	23.91737	Au	0	8.32469	49.59813
C	5.46226	4.34957	24.1468	Au	2.88376	8.32469	49.59813
H	2.89196	1.18242	24.71481	Au	5.76752	8.32469	49.59813
H	2.80985	3.71392	24.70356	Au	8.65128	8.32469	49.59813

C	3.82089	1.78796	24.75277
C	3.54143	3.17198	25.33636
O	3.08371	8.58054	25.3089
H	4.55922	1.25711	25.38528
C	3.96076	6.3364	25.40228
C	4.83566	4.01917	25.51123
H	6.47242	3.08385	25.5576
C	3.5442	7.62382	25.9483
C	4.46938	5.3202	26.19647
O	5.79196	3.27634	26.24568
H	3.10084	3.0899	26.35271
Au	11.53503	8.32469	49.59813
Au	-4.32564	10.8221	49.59813
Au	-1.44188	10.8221	49.59813
Au	1.44188	10.8221	49.59813
Au	4.32564	10.8221	49.59813
Au	7.2094	10.8221	49.59813
Au	10.09315	10.8221	49.59813
Au	-5.76752	13.31951	49.59813
Au	-2.88376	13.31951	49.59813
Au	0	13.31951	49.59813
Au	2.88376	13.31951	49.59813
Au	5.76752	13.31951	49.59813
Au	8.65128	13.31951	49.59813

Au-CB7---CPT(H)---CB7-Au:

Au	1.44188	0.83247	1.17729
Au	4.32564	0.83247	1.17729
Au	7.2094	0.83247	1.17729
Au	10.09315	0.83247	1.17729
Au	12.97691	0.83247	1.17729
Au	15.86067	0.83247	1.17729
Au	0	3.32988	1.17729
Au	2.88376	3.32988	1.17729
Au	5.76752	3.32988	1.17729
Au	8.65128	3.32988	1.17729
Au	11.53503	3.32988	1.17729
Au	14.41879	3.32988	1.17729
Au	-1.44188	5.82729	1.17729
Au	1.44188	5.82729	1.17729
Au	4.32564	5.82729	1.17729
Au	7.2094	5.82729	1.17729
Au	10.09315	5.82729	1.17729
Au	12.97691	5.82729	1.17729
Au	-2.88376	8.32469	1.17729
Au	0	8.32469	1.17729
Au	2.88376	8.32469	1.17729
Au	5.76752	8.32469	1.17729
Au	8.65128	8.32469	1.17729
Au	11.53503	8.32469	1.17729
Au	-4.32564	10.8221	1.17729
Au	-1.44188	10.8221	1.17729
Au	1.44188	10.8221	1.17729
C	4.64289	8.52747	27.51571
C	4.82428	6.16578	27.57003
H	4.95772	4.00682	27.68485
H	5.50863	9.19655	27.69146
Cl	2.26326	5.17039	27.49616
C	4.17749	6.58527	28.80203
C	4.07281	8.00527	28.80919
H	0.02317	4.32079	29.39986
O	2.01751	3.06243	29.34126
O	0.40025	6.72669	29.42834
O	1.00293	10.58271	29.4432
O	7.16223	6.13015	29.45675
H	-0.59097	8.87586	29.47976
O	6.6512	10.01809	29.49036
O	3.89167	12.07067	29.5055
H	8.20688	8.23625	29.51505
O	5.06895	3.12583	29.54751
H	7.3645	3.70012	29.62215
H	6.20655	12.41582	29.67665
H	3.45922	1.30836	29.75472
H	3.88168	4.83356	29.76974
N	3.89695	5.85993	29.89901
H	1.63927	12.90321	29.80907
C	3.54362	8.64733	29.90807
H	3.42693	9.74113	29.90773
H	-1.41385	3.57993	30.2752
H	-2.16685	9.20809	30.355

Au	4.32564	10.8221	1.17729	H	9.81199	8.43305	30.37447
Au	7.2094	10.8221	1.17729	C	-0.43894	4.11773	30.39677
Au	10.09315	10.8221	1.17729	C	-0.32683	6.59279	30.39047
Au	-5.76752	13.31951	1.17729	C	1.61311	2.76629	30.44104
Au	-2.88376	13.31951	1.17729	C	-1.08955	8.92886	30.47393
Au	0	13.31951	1.17729	C	7.80332	6.02178	30.48783
Au	2.88376	13.31951	1.17729	C	8.70844	8.29858	30.50648
Au	5.76752	13.31951	1.17729	C	7.27021	10.28274	30.50722
Au	8.65128	13.31951	1.17729	C	0.5049	10.78485	30.53773
Au	0	1.66494	3.53187	C	5.37242	2.6751	30.6415
Au	2.88376	1.66494	3.53187	C	3.96408	12.50826	30.64239
Au	5.76752	1.66494	3.53187	C	7.65976	3.56152	30.6862
Au	8.65128	1.66494	3.53187	C	6.41288	12.58005	30.75699
Au	11.53503	1.66494	3.53187	H	8.52728	2.86145	30.75641
Au	14.41879	1.66494	3.53187	C	3.41928	1.17478	30.85783
Au	-1.44188	4.16235	3.53187	C	1.55759	12.98928	30.91424
Au	1.44188	4.16235	3.53187	H	6.96721	13.53631	30.91141
Au	4.32564	4.16235	3.53187	C	3.31845	6.43507	31.00603
Au	7.2094	4.16235	3.53187	C	3.13839	7.86104	31.03954
Au	10.09315	4.16235	3.53187	N	-0.69727	5.39526	31.01678
Au	12.97691	4.16235	3.53187	N	-1.0062	7.62054	31.05594
Au	-2.88376	6.65975	3.53187	N	0.49395	3.26658	31.11007
Au	0	6.65975	3.53187	H	3.39546	0.09596	31.13002
Au	2.88376	6.65975	3.53187	N	8.4592	7.0471	31.17176
Au	5.76752	6.65975	3.53187	N	8.16891	9.4547	31.17683
Au	8.65128	6.65975	3.53187	N	8.07438	4.84393	31.17911
Au	11.53503	6.65975	3.53187	N	7.25657	11.49736	31.18585
Au	-4.32564	9.15716	3.53187	N	2.18993	1.79632	31.26547
Au	-1.44188	9.15716	3.53187	N	-0.40733	9.97088	31.20705
Au	1.44188	9.15716	3.53187	H	1.07119	13.9494	31.20179
Au	4.32564	9.15716	3.53187	N	0.71257	11.90556	31.34305
Au	7.2094	9.15716	3.53187	N	6.52378	2.96874	31.35722
Au	10.09315	9.15716	3.53187	N	4.64518	1.74799	31.37165
Au	-5.76752	11.65457	3.53187	N	5.12405	12.66303	31.3995
Au	-2.88376	11.65457	3.53187	N	2.91098	12.99396	31.41195
Au	0	11.65457	3.53187	H	-2.60847	5.04093	31.84915
Au	2.88376	11.65457	3.53187	H	-2.90824	7.48007	31.94873
Au	5.76752	11.65457	3.53187	H	9.92699	10.2852	32.01655
Au	8.65128	11.65457	3.53187	H	3.0853	4.54677	32.06495
Au	-7.2094	14.15198	3.53187	H	10.35781	6.68352	32.03747
Au	-4.32564	14.15198	3.53187	H	-0.73035	1.82272	32.07499
Au	-1.44188	14.15198	3.53187	C	2.93478	5.63524	32.10823
Au	1.44188	14.15198	3.53187	C	-1.66128	5.59122	32.07174
Au	4.32564	14.15198	3.53187	C	-1.85186	7.15364	32.11699

Au	7.2094	14.15198	3.53187	C	2.59003	8.42967	32.22184
Au	0	0	5.88645	H	-2.02076	10.89523	32.22861
Au	2.88376	0	5.88645	C	0.16762	2.45959	32.27277
Au	5.76752	0	5.88645	H	2.48976	9.52494	32.27972
Au	8.65128	0	5.88645	C	8.84874	10.13538	32.26297
Au	11.53503	0	5.88645	C	9.27188	6.55774	32.26586
Au	14.41879	0	5.88645	C	8.82806	5.05736	32.38441
Au	-1.44188	2.49741	5.88645	C	8.04264	11.4783	32.38935
Au	1.44188	2.49741	5.88645	C	-0.95481	10.60774	32.39077
Au	4.32564	2.49741	5.88645	C	1.48499	1.6219	32.49546
Au	7.2094	2.49741	5.88645	H	8.69334	12.38436	32.44492
Au	10.09315	2.49741	5.88645	H	9.68316	4.34242	32.44636
Au	12.97691	2.49741	5.88645	C	0.00398	11.83514	32.59098
Au	-2.88376	4.99482	5.88645	C	6.59104	2.25526	32.61825
Au	0	4.99482	5.88645	H	7.46289	1.55801	32.62762
Au	2.88376	4.99482	5.88645	H	1.29465	0.53857	32.69593
Au	5.76752	4.99482	5.88645	H	5.3611	14.34945	32.65883
Au	8.65128	4.99482	5.88645	C	4.88564	13.33983	32.65852
Au	11.53503	4.99482	5.88645	C	5.19627	1.52348	32.68316
Au	-4.32564	7.49222	5.88645	C	3.31576	13.3849	32.73731
Au	-1.44188	7.49222	5.88645	H	-0.53012	12.79298	32.79743
Au	1.44188	7.49222	5.88645	H	5.27483	0.43078	32.88949
Au	4.32564	7.49222	5.88645	H	2.91308	14.39256	32.99365
Au	7.2094	7.49222	5.88645	C	2.39677	6.23115	33.24635
Au	10.09315	7.49222	5.88645	C	2.22875	7.63683	33.30956
Au	-5.76752	9.98963	5.88645	N	-1.20978	5.27147	33.40503
Au	-2.88376	9.98963	5.88645	N	-1.43513	7.50661	33.45643
Au	0	9.98963	5.88645	N	0.01349	3.14991	33.52668
Au	2.88376	9.98963	5.88645	N	8.71224	9.54415	33.56654
Au	5.76752	9.98963	5.88645	N	8.97163	7.09932	33.56253
Au	8.65128	9.98963	5.88645	N	2.07078	2.25488	33.66122
Au	-7.2094	12.48704	5.88645	N	-0.81876	9.87702	33.62295
Au	-4.32564	12.48704	5.88645	N	8.06735	5.03329	33.6174
Au	-1.44188	12.48704	5.88645	N	7.31629	11.30953	33.63168
Au	1.44188	12.48704	5.88645	H	-2.05363	3.37001	33.66697
Au	4.32564	12.48704	5.88645	N	0.79048	11.44076	33.74058
Au	7.2094	12.48704	5.88645	N	4.51654	2.20578	33.76053
Au	1.44188	0.83247	8.24103	H	10.50711	8.49672	33.80302
Au	4.32564	0.83247	8.24103	N	6.59097	3.08022	33.79661
Au	7.2094	0.83247	8.24103	N	3.01313	12.43993	33.79235
Au	10.09315	0.83247	8.24103	N	5.25548	12.61559	33.84293
Au	12.97691	0.83247	8.24103	H	-2.73403	9.10138	33.89071
Au	15.86067	0.83247	8.24103	C	-1.13168	3.93246	33.94127
Au	0	3.32988	8.24103	C	9.42657	8.38263	34.05267

Au	2.88376	3.32988	8.24103	C	-1.67408	8.79683	34.06495
Au	5.76752	3.32988	8.24103	H	2.13903	5.62355	34.12841
Au	8.65128	3.32988	8.24103	H	1.8602	8.09087	34.24457
Au	11.53503	3.32988	8.24103	H	7.18188	13.33059	34.23435
Au	14.41879	3.32988	8.24103	C	-1.08454	6.3948	34.21098
Au	-1.44188	5.82729	8.24103	H	8.61541	3.12105	34.33524
Au	1.44188	5.82729	8.24103	C	1.1641	3.09302	34.29811
Au	4.32564	5.82729	8.24103	C	6.60259	12.37956	34.30235
Au	7.2094	5.82729	8.24103	C	3.25852	1.77639	34.33691
Au	10.09315	5.82729	8.24103	C	7.73035	3.8022	34.31209
Au	12.97691	5.82729	8.24103	H	3.23738	0.66178	34.3934
Au	-2.88376	8.32469	8.24103	C	8.2384	6.21312	34.33934
Au	0	8.32469	8.24103	C	7.77751	10.21023	34.34717
Au	2.88376	8.32469	8.24103	C	0.26829	10.31433	34.36345
Au	5.76752	8.32469	8.24103	C	1.71484	12.3185	34.42042
Au	8.65128	8.32469	8.24103	C	5.35264	3.09626	34.42843
Au	11.53503	8.32469	8.24103	H	1.25772	13.33005	34.5564
Au	-4.32564	10.8221	8.24103	C	4.16105	12.0288	34.46116
Au	-1.44188	10.8221	8.24103	H	-1.0918	4.02314	35.05122
Au	1.44188	10.8221	8.24103	H	-1.52563	8.67819	35.16283
Au	4.32564	10.8221	8.24103	H	9.32751	8.37523	35.16416
Au	7.2094	10.8221	8.24103	H	3.21651	2.17533	35.37618
Au	10.09315	10.8221	8.24103	H	7.49647	4.07454	35.36681
Au	-5.76752	13.31951	8.24103	H	6.53897	12.10519	35.38469
Au	-2.88376	13.31951	8.24103	O	1.33763	3.68149	35.35706
Au	0	13.31951	8.24103	O	-0.74821	6.39772	35.38634
Au	2.88376	13.31951	8.24103	H	1.87942	11.89338	35.44112
Au	5.76752	13.31951	8.24103	O	5.04631	3.75861	35.40763
Au	8.65128	13.31951	8.24103	O	0.69789	9.79216	35.38342
Au	-0.0043	1.66517	10.6489	O	7.83483	6.41605	35.47237
Au	2.88243	1.66076	10.6464	O	7.44295	9.89249	35.47753
Au	5.76819	1.66056	10.64608	O	4.20031	11.29824	35.44085
Au	8.65555	1.66474	10.64862	Au	-0.00324	-0.00532	37.61555
Au	11.53621	1.66571	10.65135	Au	2.8859	-0.02111	37.6086
Au	14.41846	1.6666	10.65192	Au	5.76699	-0.00418	37.6117
Au	-1.44622	4.16021	10.64632	Au	8.64974	0.00081	37.61069
Au	1.44258	4.16217	10.6461	Au	11.52981	0.01193	37.60865
Au	4.3256	4.15835	10.6457	Au	14.42376	0.01232	37.61081
Au	7.20865	4.16258	10.6468	Au	-1.45202	2.49198	37.62411
Au	10.09734	4.16023	10.64704	Au	1.44113	2.45708	37.71968
Au	12.97711	4.16077	10.64983	Au	4.32406	2.46768	37.7316
Au	-2.88603	6.65759	10.64985	Au	7.2195	2.47957	37.65797
Au	-0.00385	6.66095	10.64533	Au	10.10006	2.48797	37.6043
Au	2.88232	6.65948	10.64185	Au	12.97446	2.49552	37.62404

Au	5.76882	6.65951	10.64136	Au	-2.89327	4.98448	37.62503
Au	8.6545	6.66093	10.64563	Au	-0.00704	4.97703	37.70914
Au	11.53854	6.65736	10.64804	Au	2.89301	4.99794	37.67489
Au	-4.32553	9.15891	10.65446	Au	5.78942	5.01444	37.72145
Au	-1.44603	9.15808	10.64502	Au	8.6824	4.96698	37.70304
Au	1.43747	9.161	10.64467	Au	11.54329	4.98898	37.6262
Au	4.32605	9.15719	10.6429	Au	-4.32782	7.48983	37.61819
Au	7.21358	9.16049	10.64489	Au	-1.47307	7.5123	37.72181
Au	10.09717	9.15853	10.64501	Au	1.43822	7.47626	37.66159
Au	-5.76602	11.65374	10.65272	Au	4.31582	7.49418	37.62668
Au	-2.88519	11.65332	10.65226	Au	7.19919	7.52984	37.74823
Au	-0.00316	11.65693	10.64583	Au	10.11391	7.48618	37.65846
Au	2.88256	11.65609	10.64642	Au	-5.75397	9.98941	37.60831
Au	5.76846	11.65638	10.64634	Au	-2.90375	10.00334	37.613
Au	8.65474	11.65684	10.64497	Au	-0.04095	10.00628	37.77475
Au	-7.20751	14.15085	10.65225	Au	2.86514	9.97678	37.71025
Au	-4.32596	14.15044	10.648	Au	5.74699	10.00016	37.7277
Au	-1.44386	14.15067	10.65134	Au	8.70969	10.00449	37.7398
Au	1.44287	14.15544	10.64507	Au	-7.19691	12.49552	37.62
Au	4.32573	14.15734	10.6469	Au	-4.32531	12.48954	37.6289
Au	7.20883	14.15615	10.64437	Au	-1.45256	12.49926	37.61235
Au	0.00067	-0.0021	13.15332	Au	1.42933	12.50663	37.71942
Au	2.88706	-0.00472	13.15663	Au	4.32724	12.53438	37.74703
Au	5.76401	-0.00655	13.15511	Au	7.22704	12.50091	37.69405
Au	8.65123	-0.0039	13.15143	Au	-0.0106	1.66272	40.13193
Au	11.53087	0.00624	13.15547	Au	2.88138	1.6534	40.15008
Au	14.42288	0.00808	13.15143	Au	5.7767	1.65648	40.14065
Au	-1.45014	2.49454	13.17086	Au	8.65982	1.66295	40.12792
Au	1.42409	2.48435	13.11161	Au	11.53584	1.66972	40.11902
Au	4.32625	2.49135	13.10564	Au	14.41466	1.66725	40.12566
Au	7.22981	2.48284	13.10543	Au	-1.44814	4.15897	40.134
Au	10.10398	2.4927	13.16553	Au	1.44281	4.16056	40.14102
Au	12.97766	2.49727	13.1441	Au	4.32263	4.15649	40.14388
Au	-2.88765	4.99342	13.1539	Au	7.2116	4.15662	40.14425
Au	-0.017	4.98355	13.101	Au	10.10338	4.15599	40.13742
Au	2.88168	5.0013	13.12115	Au	12.97689	4.15726	40.1275
Au	5.76658	4.99739	13.12482	Au	-2.89271	6.64981	40.1325
Au	8.66765	4.97957	13.09959	Au	-0.00113	6.65945	40.13849
Au	11.54166	4.99283	13.15303	Au	2.88291	6.65801	40.14075
Au	-4.32567	7.49226	13.15906	Au	5.76197	6.6581	40.14805
Au	-1.45469	7.49388	13.13729	Au	8.66127	6.65566	40.14019
Au	1.44642	7.50723	13.1012	Au	11.54122	6.65247	40.1303
Au	4.32738	7.49436	13.14508	Au	-4.32827	9.15934	40.12374
Au	7.20603	7.49671	13.10896	Au	-1.45872	9.15695	40.157

Au	10.1064	7.49244	13.12622	Au	1.43776	9.15131	40.15113
Au	-5.76133	9.98996	13.16443	Au	4.32533	9.15273	40.14732
Au	-2.88915	9.98999	13.16433	Au	7.2188	9.16282	40.15883
Au	-0.02342	9.99457	13.11489	Au	10.10698	9.1536	40.14634
Au	2.89591	9.99808	13.10949	Au	-5.76436	11.65473	40.12147
Au	5.76314	9.99752	13.11268	Au	-2.8872	11.65767	40.12295
Au	8.67444	9.99609	13.11076	Au	-0.00937	11.66879	40.1571
Au	-7.20256	12.49035	13.15418	Au	2.87895	11.6536	40.15019
Au	-4.32595	12.48515	13.14756	Au	5.77368	11.65893	40.15336
Au	-1.44907	12.48936	13.15187	Au	8.66032	11.66691	40.14723
Au	1.43071	12.49534	13.12506	Au	-7.20633	14.1521	40.12392
Au	4.3231	12.52076	13.10118	Au	-4.32503	14.15071	40.12363
Au	7.22022	12.49738	13.12027	Au	-1.44488	14.15457	40.12027
O	0.63464	6.2353	15.53738	Au	1.44381	14.16918	40.13776
O	8.21731	5.98805	15.56528	Au	4.32449	14.17043	40.14296
O	7.49154	9.82815	15.57155	Au	7.20734	14.16359	40.1345
O	1.36199	10.09625	15.56294	Au	1.44188	0.83247	42.5344
O	2.63677	3.08038	15.57159	Au	4.32564	0.83247	42.5344
O	4.44232	11.75796	15.58888	Au	7.2094	0.83247	42.5344
O	6.14566	2.91999	15.57902	Au	10.09315	0.83247	42.5344
H	9.17522	8.17143	15.63994	Au	12.97691	0.83247	42.5344
H	-0.32653	8.43335	15.65215	Au	15.86067	0.83247	42.5344
H	0.38921	3.82482	15.65841	Au	0	3.32988	42.5344
H	8.46263	3.54418	15.78545	Au	2.88376	3.32988	42.5344
H	6.76893	12.17138	15.79406	Au	5.76752	3.32988	42.5344
H	2.14646	12.40601	15.89315	Au	8.65128	3.32988	42.5344
H	4.34335	1.35445	15.9064	Au	11.53503	3.32988	42.5344
C	-0.00836	6.14002	16.57402	Au	14.41879	3.32988	42.5344
H	10.7114	8.45297	16.55073	Au	-1.44188	5.82729	42.5344
C	8.8223	5.91662	16.62568	Au	1.44188	5.82729	42.5344
C	8.03047	10.12496	16.62876	Au	4.32564	5.82729	42.5344
H	-1.82006	8.75772	16.61763	Au	7.2094	5.82729	42.5344
C	0.90469	10.3261	16.67313	Au	10.09315	5.82729	42.5344
C	9.61721	8.24936	16.65966	Au	12.97691	5.82729	42.5344
C	2.3267	2.68926	16.68744	Au	-2.88376	8.32469	42.5344
C	-0.73576	8.49271	16.68604	Au	0	8.32469	42.5344
C	0.07167	3.67238	16.71487	Au	2.88376	8.32469	42.5344
C	6.35046	2.60628	16.74202	Au	5.76752	8.32469	42.5344
C	4.48291	12.13579	16.75005	Au	8.65128	8.32469	42.5344
H	-0.81799	2.99755	16.72325	Au	11.53503	8.32469	42.5344
C	8.67393	3.4524	16.87476	Au	-4.32564	10.8221	42.5344
C	6.93439	12.32865	16.88663	Au	-1.44188	10.8221	42.5344
C	2.05131	12.49956	17.00061	Au	1.44188	10.8221	42.5344
C	4.30552	1.23949	17.01379	Au	4.32564	10.8221	42.5344

H	9.53427	2.75514	17.01303	Au	7.2094	10.8221	42.5344
H	7.41866	13.3228	17.03949	Au	10.09315	10.8221	42.5344
N	-0.30507	4.95964	17.242	Au	-5.76752	13.31951	42.5344
H	1.57557	13.47861	17.24361	Au	-2.88376	13.31951	42.5344
H	4.33003	0.15293	17.26141	Au	0	13.31951	42.5344
N	-0.59372	7.18458	17.28113	Au	2.88376	13.31951	42.5344
N	9.41636	6.9738	17.30933	Au	5.76752	13.31951	42.5344
N	8.96918	9.36228	17.31712	Au	8.65128	13.31951	42.5344
N	7.83832	11.30155	17.34179	Au	0	0	44.88897
N	9.0577	4.7578	17.35773	Au	2.88376	0	44.88897
N	-0.01151	9.54174	17.36831	Au	5.76752	0	44.88897
N	1.17893	3.03536	17.39762	Au	8.65128	0	44.88897
N	1.18212	11.44058	17.45583	Au	11.53503	0	44.88897
N	3.04557	1.78675	17.46076	Au	14.41879	0	44.88897
N	7.49306	2.88296	17.48525	Au	-1.44188	2.49741	44.88897
N	5.63029	12.32202	17.51205	Au	1.44188	2.49741	44.88897
N	3.39264	12.48093	17.53619	Au	4.32564	2.49741	44.88897
N	5.48801	1.87566	17.55273	Au	7.2094	2.49741	44.88897
H	10.605	10.37813	18.18526	Au	10.09315	2.49741	44.88897
H	11.30445	6.62909	18.19454	Au	12.97691	2.49741	44.88897
H	-2.46831	6.96565	18.21772	Au	-2.88376	4.99482	44.88897
H	-2.04136	4.5506	18.35395	Au	0	4.99482	44.88897
C	-1.39248	6.71574	18.39345	Au	2.88376	4.99482	44.88897
H	-1.56619	10.53225	18.39957	Au	5.76752	4.99482	44.88897
C	-1.11212	5.16772	18.41697	Au	8.65128	4.99482	44.88897
C	9.55138	10.09733	18.42572	Au	11.53503	4.99482	44.88897
C	10.21793	6.51309	18.42799	Au	-4.32564	7.49222	44.88897
H	0.17419	1.52898	18.48423	Au	-1.44188	7.49222	44.88897
C	-0.50748	10.21238	18.55637	Au	1.44188	7.49222	44.88897
C	8.59405	11.33384	18.56545	Au	4.32564	7.49222	44.88897
C	9.76926	5.01733	18.58219	Au	7.2094	7.49222	44.88897
C	1.02662	2.24264	18.60293	Au	10.09315	7.49222	44.88897
H	9.13991	12.30561	18.65195	Au	-5.76752	9.98963	44.88897
H	10.62441	4.30656	18.68659	Au	-2.88376	9.98963	44.88897
C	0.48903	11.4149	18.71686	Au	0	9.98963	44.88897
C	2.41969	1.52736	18.73122	Au	2.88376	9.98963	44.88897
C	5.3452	12.91807	18.80059	Au	5.76752	9.98963	44.88897
C	7.4646	2.28505	18.79952	Au	8.65128	9.98963	44.88897
C	6.06984	1.5569	18.83685	Au	-7.2094	12.48704	44.88897
H	5.79532	13.94039	18.85096	Au	-4.32564	12.48704	44.88897
C	3.77147	12.92982	18.85327	Au	-1.44188	12.48704	44.88897
H	-0.02364	12.39328	18.88974	Au	1.44188	12.48704	44.88897
H	2.33548	0.42523	18.89862	Au	4.32564	12.48704	44.88897
H	8.3298	1.5884	18.92341	Au	7.2094	12.48704	44.88897

H	6.15042	0.44841	18.95348	Au	0	1.66494	47.24355
H	3.34485	13.94505	19.04466	Au	2.88376	1.66494	47.24355
N	-0.46427	4.96594	19.6947	Au	5.76752	1.66494	47.24355
N	-0.98831	7.14619	19.70626	Au	8.65128	1.66494	47.24355
N	9.91887	7.0766	19.71409	Au	11.53503	1.66494	47.24355
N	9.48042	9.48445	19.72214	Au	14.41879	1.66494	47.24355
N	7.87207	11.047	19.79116	Au	-1.44188	4.16235	47.24355
N	8.97955	5.04214	19.80095	Au	1.44188	4.16235	47.24355
N	-0.3772	9.50793	19.80197	Au	4.32564	4.16235	47.24355
N	0.90755	2.95642	19.8476	Au	7.2094	4.16235	47.24355
H	11.35556	8.57888	19.87275	Au	10.09315	4.16235	47.24355
N	1.26344	11.0354	19.8815	Au	12.97691	4.16235	47.24355
N	3.00436	2.16797	19.88881	Au	-2.88376	6.65975	47.24355
N	7.40179	3.19857	19.91804	Au	0	6.65975	47.24355
N	3.47012	12.04362	19.9534	Au	2.88376	6.65975	47.24355
N	5.71048	12.15358	19.96235	Au	5.76752	6.65975	47.24355
N	5.43249	2.13345	19.99065	Au	8.65128	6.65975	47.24355
H	-2.30182	8.74147	20.04798	Au	11.53503	6.65975	47.24355
H	-1.16062	3.02838	20.1119	Au	-4.32564	9.15716	47.24355
C	10.299	8.38899	20.17361	Au	-1.44188	9.15716	47.24355
C	-1.24412	8.4521	20.26054	Au	1.44188	9.15716	47.24355
C	-0.26322	3.66808	20.29458	Au	4.32564	9.15716	47.24355
H	7.54734	13.02157	20.44435	Au	7.2094	9.15716	47.24355
C	7.04851	12.02046	20.47392	Au	10.09315	9.15716	47.24355
C	8.56193	3.84183	20.48963	Au	-5.76752	11.65457	47.24355
H	9.40485	3.10612	20.49981	Au	-2.88376	11.65457	47.24355
C	-0.5017	6.10816	20.50219	Au	0	11.65457	47.24355
C	9.15921	6.22782	20.51857	Au	2.88376	11.65457	47.24355
C	8.48738	10.03258	20.53701	Au	5.76752	11.65457	47.24355
H	4.18369	0.56198	20.55952	Au	8.65128	11.65457	47.24355
C	4.18734	1.67953	20.55071	Au	-7.2094	14.15198	47.24355
C	2.16678	11.93424	20.55953	Au	-4.32564	14.15198	47.24355
C	0.66422	9.99155	20.5964	Au	-1.44188	14.15198	47.24355
H	1.70398	12.95167	20.62653	Au	1.44188	14.15198	47.24355
C	2.07007	2.91325	20.61709	Au	4.32564	14.15198	47.24355
C	6.20831	3.09717	20.62699	Au	7.2094	14.15198	47.24355
C	4.61041	11.66603	20.67087	Au	1.44188	0.83247	49.59813
H	10.20511	8.37003	21.28156	Au	4.32564	0.83247	49.59813
H	-1.07907	8.35684	21.35716	Au	7.2094	0.83247	49.59813
H	-0.13126	3.84974	21.38504	Au	10.09315	0.83247	49.59813
H	6.9588	11.66405	21.52522	Au	12.97691	0.83247	49.59813
H	8.32097	4.13023	21.53784	Au	15.86067	0.83247	49.59813
H	2.31961	11.51403	21.57962	Au	0	3.32988	49.59813
H	4.1392	2.06244	21.59104	Au	2.88376	3.32988	49.59813

O	5.90735	3.70328	21.64146	Au	5.76752	3.32988	49.59813
O	8.77583	6.47063	21.64779	Au	8.65128	3.32988	49.59813
O	-0.21101	6.17389	21.68186	Au	11.53503	3.32988	49.59813
O	8.24249	9.71983	21.68711	Au	14.41879	3.32988	49.59813
O	0.96068	9.60765	21.71221	Au	-1.44188	5.82729	49.59813
O	4.63714	11.06858	21.7294	Au	1.44188	5.82729	49.59813
O	2.22594	3.389	21.72733	Au	4.32564	5.82729	49.59813
H	5.99553	5.74527	22.91965	Au	7.2094	5.82729	49.59813
H	5.85241	1.3996	23.29663	Au	10.09315	5.82729	49.59813
H	7.32449	6.76236	23.54932	Au	12.97691	5.82729	49.59813
H	4.09938	1.10579	23.58972	Au	-2.88376	8.32469	49.59813
H	4.60221	3.59059	23.63109	Au	0	8.32469	49.59813
O	7.61752	4.72671	23.70198	Au	2.88376	8.32469	49.59813
C	6.70783	5.86853	23.7656	Au	5.76752	8.32469	49.59813
C	5.03506	1.49311	24.03975	Au	8.65128	8.32469	49.59813
O	8.07405	2.61862	24.23017	Au	11.53503	8.32469	49.59813
C	7.32321	3.58527	24.33787	Au	-4.32564	10.8221	49.59813
C	4.84771	2.94228	24.49396	Au	-1.44188	10.8221	49.59813
H	5.28481	0.84356	24.90086	Au	1.44188	10.8221	49.59813
O	5.94602	8.398	25.0214	Au	4.32564	10.8221	49.59813
C	6.03079	5.98677	25.0909	Au	7.2094	10.8221	49.59813
H	4.00033	3.02046	25.20706	Au	10.09315	10.8221	49.59813
C	6.09284	3.48869	25.25614	Au	-5.76752	13.31951	49.59813
C	5.70978	7.3227	25.58184	Au	-2.88376	13.31951	49.59813
C	5.77839	4.8453	25.83913	Au	0	13.31951	49.59813
H	7.17524	2.0684	25.90496	Au	2.88376	13.31951	49.59813
O	6.43505	2.58124	26.30371	Au	5.76752	13.31951	49.59813
N	5.07633	7.29926	26.84294	Au	8.65128	13.31951	49.59813
H	3.90356	9.07575	26.89569				
C	5.18846	4.90749	27.11251				

References

[1] A. Day, A. P. Arnold, R. J. Blanch, B. Snushall, *J. Org. Chem.* **2001**, *66*, 8094-8100.

[2] B. Xiao, F. Liang, S. Liu, J. Im, Y. Li, J. Liu, B. Zhang, J. Zhou, J. He, S. Chang, *Nanotechnology* **2018**, *29*, 365501.

[3] M. R. di Nunzio, B. Cohen, A. Douhal, *J. Phys. Chem. A* **2011**, *115*, 5094-5104.

[4] K. Gavvala, A. Sengupta, P. Hazra, *ChemPhysChem* **2013**, *14*, 532-542.

[5] J. Fassberg, V. J. Stella, *J. Pharm. Sci.* **1992**, *81*, 676-684.

[6] N. Dong, S.-F. Xue, Q.-J. Zhu, Z. Tao, Y. Zhao, L.-X. Yang, *Supramol. Chem.* **2008**, *20*, 663-671.

[7] M. Vlčková, P. Barták, V. Kubáň, *J. Chromatogr. A* **2004**, *1040*, 141-145.

[8] M. A. Alnajjar, W. M. Nau, A. Hennig, *Org. Biomol. Chem.* **2021**, *19*, 8521-8529.
